# The Application of Metal–Organic Frameworks as Drug Delivery Systems: From the Perspective of Molecular Dynamics Simulations

**DOI:** 10.1002/mco2.70692

**Published:** 2026-03-28

**Authors:** Jiahao Xu, Hanzi Zheng, Yue Gao, Yuanqiu Lai, Mengya Peng, Yike Hu, Tianmeng Yuan, Xiang Liu, Shihan Zhou, Wei Duan, Jia‐Wei Shen, Yongke Zheng

**Affiliations:** ^1^ Zhejiang Provincial Key Laboratory of Anti‐Cancer Chinese Medicines and Natural Medicines School of Pharmacy Hangzhou Normal University Hangzhou Zhejiang China; ^2^ Department of Rehabilitation Affiliated Hangzhou First People's Hospital School of Medicine Westlake University Hangzhou Zhejiang China; ^3^ Department of Intensive Care Unit Hangzhou Geriatric Hospital Hangzhou China; ^4^ Engineering Laboratory of Development and Application of Traditional Chinese Medicines Collaborative Innovation Center of Traditional Chinese Medicines of Zhejiang Province Hangzhou Normal University Hangzhou Zhejiang China

**Keywords:** drug delivery systems, force field development, host–guest interactions, metal–organic frameworks, molecular dynamics simulation, multiscale computational modeling

## Abstract

Metal–organic frameworks (MOFs) have emerged as a promising class of nanomaterials for drug delivery due to their exceptionally high surface area, tunable pore structures, and chemical versatility. However, conventional experimental techniques cannot fully capture atomic‐scale drug–carrier interactions or transient diffusion processes within MOF pores. Molecular dynamics (MD) simulation, a computational technique that tracks atom‐level movements over time, has thus become indispensable for probing these microscopic mechanisms. This review introduces the fundamentals of MD simulation and comprehensively examines how MD simulation reveals drug adsorption mechanisms, functionalization effects, and release kinetics in MOF‐based delivery systems. Then, it systematically compares major MOF families including isoreticular metal–organic frameworks, zeolitic imidazolate frameworks, Materials of Institute Lavoisier Frameworks, University of Oslo Frameworks, and porous coordinated networks and highlight their distinct host–guest interactions and stimuli‐responsive behaviors. The integration of multiscale modeling and machine learning further enhances predictive capabilities for carrier design. By establishing MD simulation as a fundamental tool for understanding nanoscale drug–carrier interactions, this review provides a theoretical foundation for developing efficient, stable, and responsive MOF‐based nanocarriers, advancing the field of precision nanomedicine.

## Graphical abstract

This review article provides a comprehensive exploration of the synergistic integration of molecular dynamics (MD) simulations and metal–organic frameworks (MOFs) to advance the design and optimization of nanoscale drug delivery systems (DDSs). We believe this work will be of significant interest to researchers in nanomedicine, computational biology, and pharmaceutical sciences, as it bridges cutting‐edge computational methodologies with experimental applications in precision drug delivery.

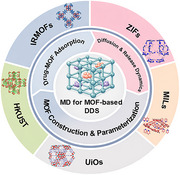

## 1 Introduction

In recent decades, nanomedicine has made significant progress and have been studied in preclinical or clinical treatments [1], including diagnostic [2] and therapeutic [3] procedures. Until now, a variety of nanomaterials have been developed for use as diagnostic and therapeutic agents in biomedicine. These nanomaterials have adjustable sizes, unique surface characteristics, and high loading efficiency, offering advantages such as controlled release, high accumulation, and the ability to promote blood circulation [4, 5, 6]. These advantages enhance therapeutic efficacy and mitigate adverse effects [7]. Although some nanocarriers currently in development still have limitations, such as low drug‐carrying efficiency, high toxicity, and poor biocompatibility, researchers are working on a new generation of nanocarrier materials that have low toxicity, high drug‐loading capacity, and good biocompatibility. With continued advances in materials science and drug delivery technologies, these new materials show promise for improving drug delivery [8]. Currently, nanomaterials are mainly categorized into inorganic and organic types. Inorganic nanomaterials include metal‐based, metal oxide‐based, and carbon‐based nanomaterials, while organic nanomaterials include polymer nanoparticles, liposomes, micelles, dendrimers, and so on. These types of nanomaterials are crucial in nanomedicine and provide novel opportunities for clinical treatments. The field of nanomedicine has made significant advances, but concerns remain regarding the long‐term safety of these materials, including their behaviors in the human body and the environment. To ensure the successful application of nanotechnology in medicine, it is necessary to establish effective regulatory strategies and clinical evaluations [9].

**FIGURE 1:**
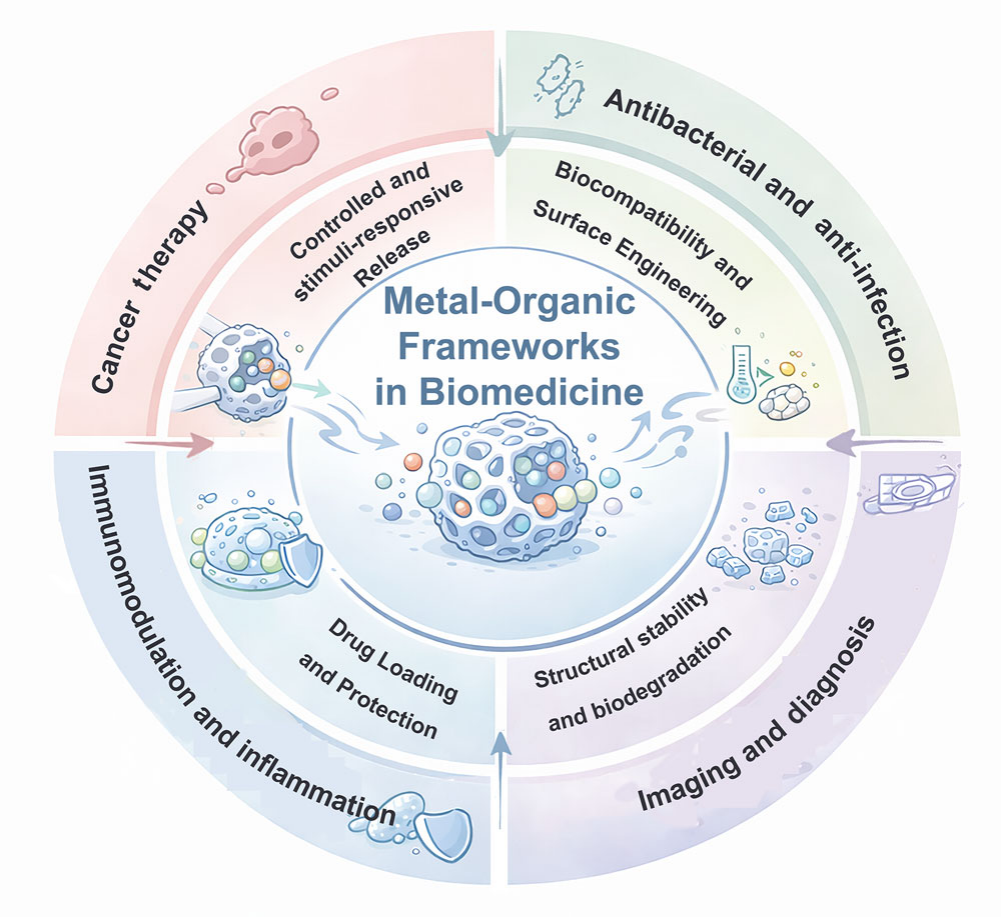
Schematic overview of functional properties and biomedical application domains of metal–organic frameworks (MOFs) in drug delivery systems. MOFs serve as versatile porous nanoplatforms capable of encapsulating and protecting therapeutic or diagnostic cargos, while their tunable structures enable drug loading, structural stability, controllable biodegradation, and surface engineering for improved biocompatibility and stimuli‐responsive release, supporting diverse biomedical applications including cancer therapy, antibacterial treatment, immunomodulation, and imaging and diagnosis.

Metal–organic frameworks (MOFs), also known as porous coordination polymers, have gained significant attention in recent years as a new class of nanoscale drug delivery systems (DDSs) [10]. MOFs are porous crystalline materials composed of metal ions or metal clusters with organic linkers. They are known for their extremely high specific surface area, with some of them reaching 10,000 m^2^/g [11]. Currently, the applications of MOFs have been extended to various fields, including sensing [12], gas adsorption [13] and separation [14], biomass conversion [15], and heterogeneous catalysis [16]. Due to their unique optical properties and X‐ray attenuation, MOFs are also used in various molecular imaging techniques such as fluorescence imaging [17], electron computed tomography [18], magnetic resonance imaging [19], and positron emission computed tomography [20]. To provide an overview of the biomedical relevance of MOFs, Figure 1 summarizes the key functional roles and application domains of MOFs in biomedicine. At the core, MOFs serve as versatile porous platforms capable of encapsulating and protecting diverse therapeutic or diagnostic cargos. Their highly tunable structures enable efficient drug loading, enhanced structural stability, and controllable biodegradation, which are critical for maintaining cargo integrity and regulating in vivo fate. Surrounding these intrinsic material properties, MOFs can be further engineered through surface modification and compositional design to improve biocompatibility and achieve controlled, stimuli‐responsive release behaviors. Such capabilities underpin a wide range of biomedical applications, including cancer therapy, antibacterial and anti‐infective treatments, immunomodulation and inflammation regulation, as well as imaging and diagnostic applications. Despite the promising applications of MOFs, they have some drawbacks, such as chemical stability issues, which limit their potential uses. However, researchers are addressing these limitations and enhancing MOFs’ physical and chemical properties by modifying their multifunctionality and structural plasticity. They are also combining MOFs with other functional materials, such as metal nanoparticles, graphene, and carbon nanotubes, to introduce properties such as optics, electricity, magnetism, and catalysis [21]. Therefore, the study of utilizing MOFs as drug carriers is currently a popular research topic, with a focus on translational and clinical applications. Despite the remarkable progress achieved through experimental investigations, most studies on MOF‐based DDSs still face inherent limitations in elucidating the molecular mechanisms underlying drug loading and release. Experimental techniques such as spectroscopy, adsorption isotherms, and microscopy primarily provide macroscopic or averaged structural information, which makes it challenging to capture the atomic‐level interactions, dynamic conformational changes, and diffusion pathways occurring within MOF pores. Moreover, the transient and nanoscale nature of drug–MOF interactions often exceed the temporal and spatial resolution of conventional characterization methods [22, 23].

Recently, molecular dynamics (MD) simulation has emerged as an indispensable computational approach to complement experiments, enabling real‐time visualization of host–guest interactions, energy landscapes, and the mechanistic origins of controlled drug release [24]. This provides atomistic insight into the adsorption, diffusion, and release behavior of drug molecules in MOFs under various physiological conditions [25]. MD simulation is a computer‐based technique that utilizes Newtonian mechanics to elucidate the macroscopic properties of matter by tracking the microscopic motion of molecular systems [26]. The technique was first introduced by Alder et al. [27] in 1957, who studied rigid spherical molecular systems. Its initial application to protein systems (bovine trypsin inhibitors) followed in 1977 [28], and it was extended to nonequilibrium systems by Gillan et al. [29] in 1983. The foundation of MD simulation is the construction of force field models, which utilize mathematical functions to describe interparticle interactions (including bond lengths, bond angles, van der Waals, and Coulomb forces, amongst others) [30]. These models are then employed to calculate the conformational integral of the system, thereby deriving the thermodynamic parameters [31, 32]. A unique advantage of MD simulation is its capacity to simulate MD under varying experimental conditions (pH, temperature, and solvent environment), thereby presenting microscopic mechanisms (e.g., protein conformational changes and biomolecule interactions) in both temporal and spatial dimensions [33]. The enhancement of computational capabilities and the refinement of algorithms have contributed to the emergence of MD simulation as a significant research instrument within the domains of chemical physics, materials science and biomedicine. In the domain of drug research and development, it has the capacity to dynamically demonstrate drug dissolution, controlled release behavior, and targeted drug delivery processes [34, 35, 36]. In addition, it can accurately calculate binding free energy, conformational stability, and other energy data. This provides a key basis for understanding drug action mechanisms and optimizing molecular structure [37]. The technology is of outstanding value for the characterization of biomolecules such as membrane permeability, lipid–protein interactions, protein–ligand binding, which can both complement experimental data and predict the nature of complex chemical systems [38]. At present, MD simulation has been employed at every stage of the drug design process, playing an irreplaceable role in the analysis of disease mechanisms and the screening of lead compounds. With continuous improvements in hardware performance and force field models, this technology, which combines environmental controllability and microscopic analytical power, is poised to yield more breakthrough applications in the pharmaceutical industry [39].

Although numerous experimental and theoretical studies have explored MOF‐based DDSs, a systematic understanding of how MD simulation elucidates drug–MOF interactions remain limited [40, 41]. Existing reviews typically focus on the synthesis, structure, and pharmacological applications of MOFs, yet few have comprehensively discussed how MD contributes to unveiling adsorption mechanisms, release kinetics, and stability at the atomic level [42, 43]. As MD techniques continue to evolve, incorporating coarse‐grained models, reactive force fields, and machine learning (ML) integration, it becomes increasingly necessary to summarize current advances and identify future research opportunities in this interdisciplinary domain. Therefore, this review aims to systematically summarize the applications of MD simulation in elucidating the drug‐loading, interaction, and release mechanisms in MOF‐based DDSs. Compared with previous reviews focusing on either MOF synthesis or general biomedical applications, this article specifically highlights how MD simulation provides quantitative and predictive understanding that guides rational material design. This review is organized as follows: Section 2 introduces the fundamental concepts of MD simulation applied to MOFs, including basic theoretical principles, force‐field development, and commonly used analytical approaches. Section 3 summarizes MD studies on drug‐loading behavior and host–guest interactions, highlighting how molecular‐level interactions govern adsorption and retention within MOF pores. Section 4 focuses on diffusion processes and release dynamics, emphasizing the relationship between framework structure, environmental conditions, and transport behavior. Section 5 provides a comparative overview of representative MOF families, elucidating how differences in composition, topology, and stability influence drug delivery performance. Building on these sections, Section 6 integrates molecular insights derived from MD simulation to extract general mechanistic principles relevant to biological interactions and translational considerations, while also discussing emerging computational strategies that extend beyond conventional MD. Finally, Section 7 outlines current challenges and future perspectives in the field. Through this structured framework, the review aims to bridge atomistic simulation results with experimentally observed behaviors, offering a coherent molecular‐level perspective on MOF‐based DDSs.

## 2 Fundamentals of MD Simulations for MOFs

The application of MD simulation to the study of MOFs has demonstrated significant advantages in the field of nanodrug delivery and functional materials, and MD simulation can resolve the structural stability, pore flexibility, molecular adsorption behavior, and guest diffusion kinetics in the pore channels of MOF materials at the atomic level, thus providing an in‐depth understanding of their structure–function relationships. Compared with traditional experimental methods, MD simulation is characterized by controllability, high resolution, low cost, good reproducibility, and is capable of rapidly predicting the behavior and properties of MOFs in different environments without relying on a large number of experimental reagents and operating conditions [44]. It is particularly suitable for studying the interaction, loading process and release mechanism of MOF–drugs/biomolecules (e.g., proteins, DNA, lipids, etc.) complexes to reveal the mechanism of action at the molecular level. In addition, with the help of coarse‐grained models or reactive force fields, MD simulation can be extended to more complex systems, such as the dynamic response of MOFs under physiological conditions, biofilm penetration behavior, or degradation processes, providing theoretical support and mechanism prediction for MOF design and performance optimization. Therefore, MD simulation has become one of the key tools in MOF materials research linking microstructure regulation and macroproperty investigation.

### 2.1 Basic Principles and Workflow of MD

**FIGURE 2:**
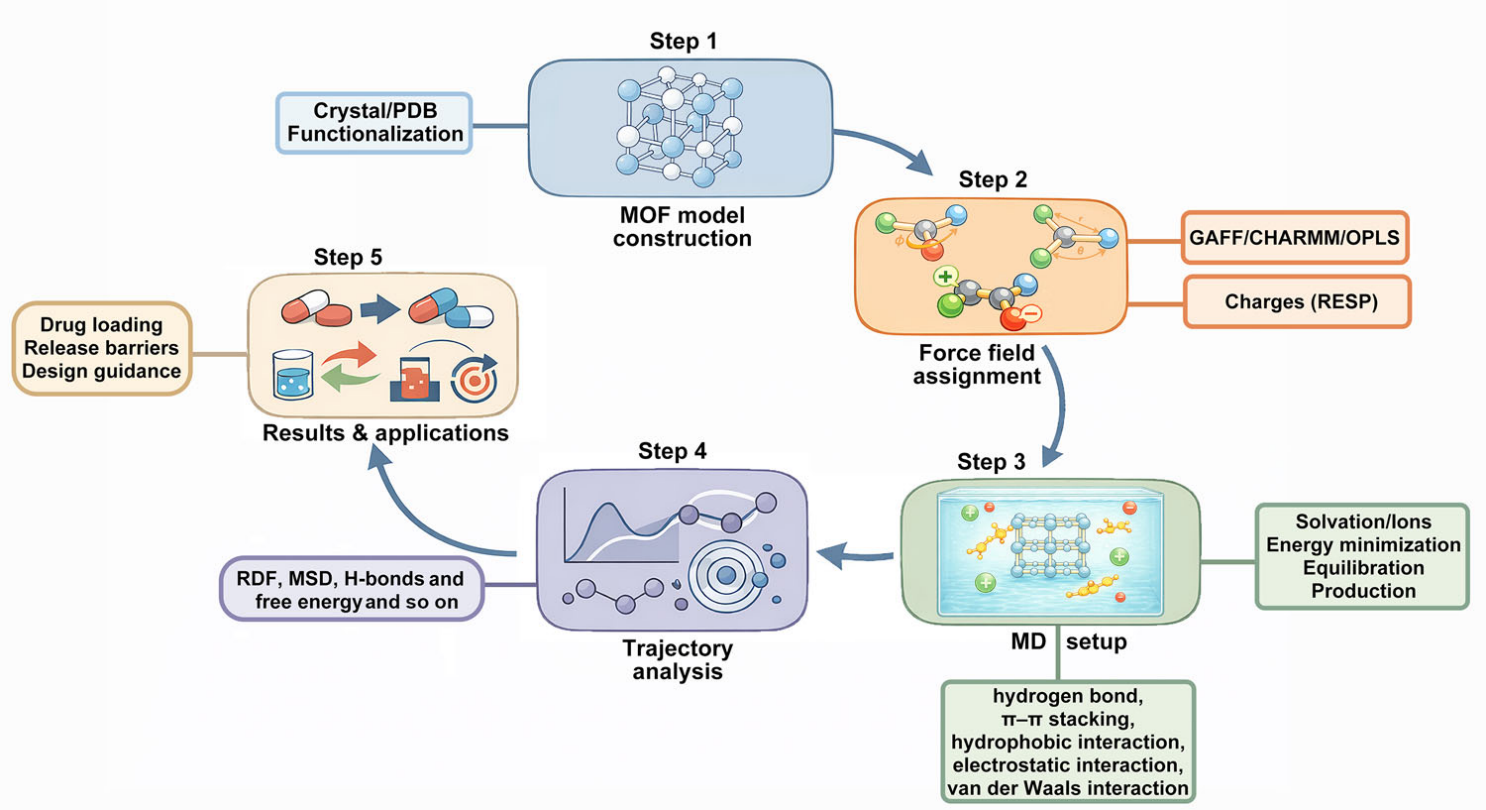
The workflow of molecular dynamics simulation for MOF‐based drug delivery systems. MD simulations can be implemented through stepwise procedures, including MOF model construction and functionalization, force‐field assignment and charge calculation, system setup with solvation and equilibration, production simulations, trajectory analysis using RDF, MSD, hydrogen‐bond and free‐energy evaluation, and translation of these results into guidance for drug loading and release optimization.

The MD simulation workflow for MOF‐based DDSs generally involves four major stages: model construction, force field assignment, simulation setup, and trajectory analysis. A schematic representation of the typical MD workflow applied to MOF‐based DDSs is shown in Figure 2. In the first step, structural models of the MOF framework and guest molecules are constructed based on crystallographic data or experimentally optimized geometries. Drug molecules are inserted into the MOF pores according to adsorption or encapsulation configurations determined from preliminary docking or self‐assemble. In the second step, appropriate force fields are assigned to describe interatomic interactions, including bonded terms (bond stretching, angle bending, torsion) and nonbonded terms (electrostatics and van der Waals interactions). The third stage involves simulation setup and execution, where the system is solvated, energy‐minimized, and equilibrated under controlled temperature and pressure using ensembles such as isothermal‐isobaric (NPT) ensemble or canonical (NVT) ensemble. The production MD run then propagates atomic motions according to Newton's equations of motion, generating trajectories that capture temporal evolution at femtosecond–nanosecond timescales. Finally, trajectory analysis provides quantitative insight into drug–MOF interactions through radial distribution functions (RDFs), hydrogen‐bond occupancy, diffusion coefficients, and binding free energy profiles. These analyses bridge microscopic interactions with macroscopic behavior such as adsorption affinity and release kinetics.

In the domain of molecular simulation, the progressive development of computational methodologies has led to a spectrum of modeling approaches that span multiple time and length scales. At the macroscopic level, continuum‐based models treat drug diffusion and transport as continuous processes governed by averaged concentration gradients and bulk properties [45]. While such descriptions are effective for large‐scale systems, their underlying assumptions become increasingly invalid as the characteristic dimensions approach the nanometer regime. For nanoscale drug delivery platforms such as MOFs, molecular discreteness, interfacial effects, and local heterogeneities play a dominant role in determining transport behavior and interaction mechanisms [46].

**FIGURE 3:**
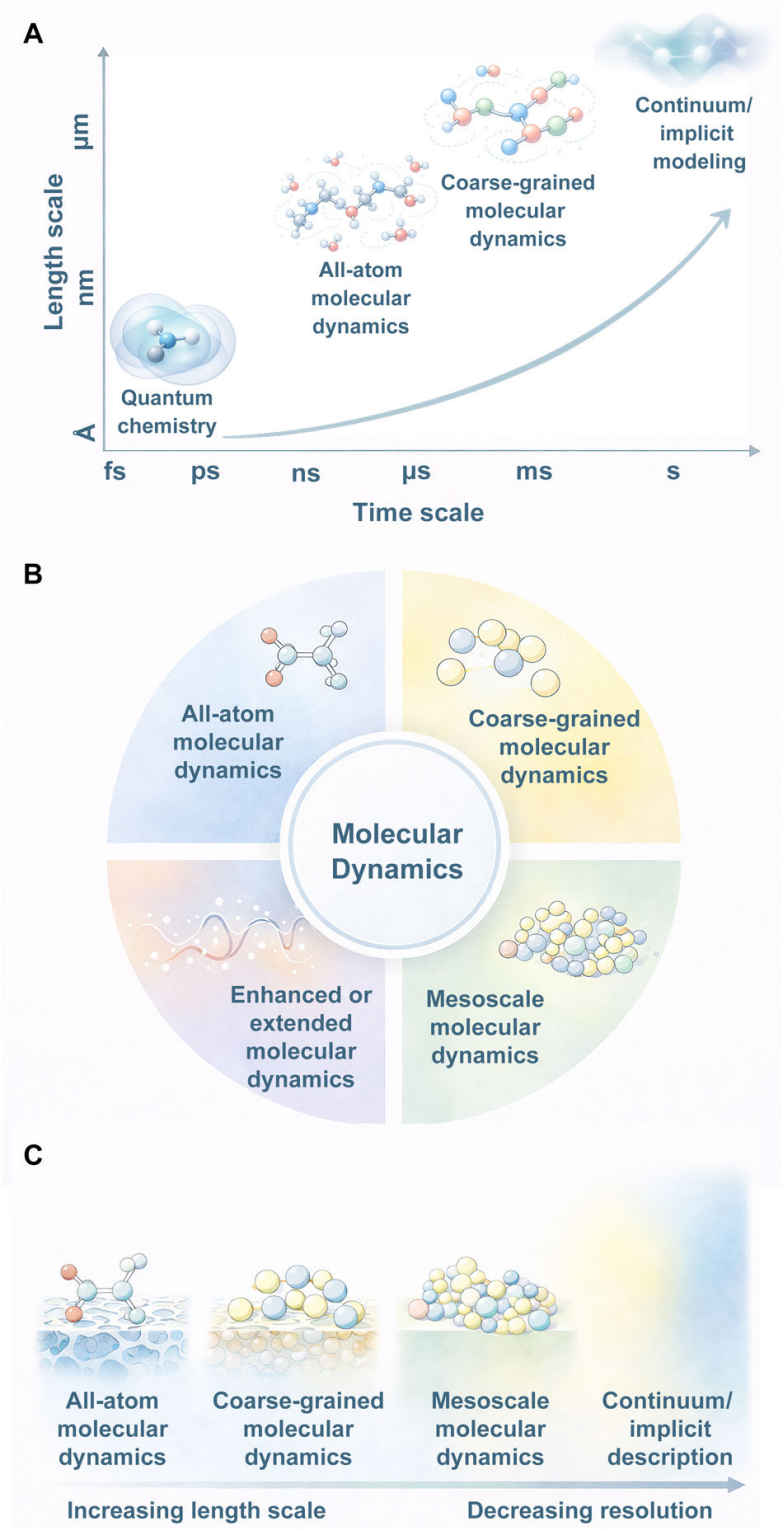
Multiscale molecular dynamics methodologies across time and length scales. (A) Time and length scales covered by different molecular simulation approaches; (B) classification of molecular dynamics methodologies according to modeling resolution; (C) applicable computational scales of representative molecular dynamics methods from atomistic to coarse‐grained descriptions.

Under these conditions, continuum approaches are unable to capture stochastic dynamics, site‐specific interactions, and transient structural fluctuations, necessitating the use of molecular‐level simulation techniques. MD simulation provides a versatile framework to bridge this gap; however, MD itself encompasses a hierarchy of methods with distinct resolutions and accessible scales. As summarized in Figure 3, atomistic simulations offer detailed descriptions of drug–MOF interactions at short time and length scales, whereas coarse‐grained and mesoscale MD approaches extend simulations toward longer times and larger system sizes by reducing molecular resolution. At the upper end of this hierarchy, continuum or implicit representations sacrifice microscopic detail to describe collective transport behavior over extended spatial and temporal domains. This multiscale perspective highlights that no single simulation method is universally applicable. Instead, the selection of an appropriate computational approach must be guided by the specific physical processes of interest, balancing molecular resolution against accessible time and length scales.

In multiscale simulations, coarse‐grained MD (CG‐MD) and all‐atom MD (AA‐MD) methods are regarded as the most pragmatic instruments in the examination of DDSs. These models are notable for their ability to simultaneously consider computational efficiency and system scale while preserving molecular structural characteristics. Consequently, they are well suited for simulating the interactions between drugs and nanocarriers within complex biological systems [47, 48]. These methods have been extensively employed to investigate the physical–chemical interaction mechanisms between nanoparticles and the extracellular matrix, cell membranes, and biological macromolecules. This provides significant theoretical underpinnings for the rational design and performance enhancement of nanodrug systems.

MD simulation is based on classical Newtonian mechanical equations, which are utilized to calculate the position and velocity of each atom in the system at different time steps. This process provides trajectories of molecular motion and describes the dynamics within the system. The total potential energy of the molecules in the system is a function of the position of each atom in the molecule, *U*(*r*). Therefore, the force *F_i_* exerted on an atom *i* of mass *m_i_* is given by:

(1) $$F_{i}=-\nabla_{i}U=\frac{dU}{dr_{i}}\;$$

According to Newton's law, if *v_i_* denotes the velocity vector of the atom and *r_i_* denotes the position vector of the atom, then the acceleration of atom *i* at that position is:

(2) $$a_{i}=\frac{F_{i}}{m_{i}}=\frac{dv_{i}}{dt}=\frac{d^{2}r_{i}}{dt^{2}}\;$$

After a time interval Δ*t*, the velocity and position of the atom *i* are:

(3) $$v_{i}\;\left(t+\Delta t\right)=v_{i}\;\left(t\right)+\frac{F_{i}\left(t\right)}{m_{i}}\Delta t$$

(4) $$r_{i}\;\left(t+\Delta t\right)=r_{i}\;\left(t\right)+v_{i}\left(t\right)\Delta t+\frac{1}{2}\frac{F_{i}\left(t\right)}{m_{i}}\Delta t^{2}$$

where *v_i_* (*t*) and *v_i_* (*t* + Δ*t*) denote the velocity of the atom at moment *t* and *t* + Δ*t*, respectively, and *r_i_* (*t*) and *r_i_* (*t* + Δ*t*) denote the position of the atom at moment *t* and *t* + Δ*t*, respectively. *F_i_* (*t*) is the force on the atom at moment *t*.

AA‐MD simulation is capable of modeling molecular behavior in the tens of nanometers range on nanosecond to millisecond time scales by explicitly representing each atom in the system [49, 50]. Interactions between particles are defined by a potential energy function (i.e., a force field) that includes both bonding terms (bond lengths, bond angles, dihedral angles, and improper dihedral angles) and nonbonding terms (electrostatic and van der Waals interactions). Commonly used all‐atom force fields include OPLS, CHARMM, and AMBER, which are widely used to model the interactions between nanomaterials and biomolecules such as carbohydrates, nucleic acids, proteins, and lipids [49, 51]. Although all‐atom force fields provide highly accurate structural and dynamical descriptions, they are often limited by the lack of experimental data when modeling nanoparticle surfaces. Therefore, more adaptable and specialized force fields are required for different research systems, such as the COMPASS force field [52] commonly used for materials systems, the ff19SB force field [53] for protein systems, the lipid21 force field [54] for lipid bilayers, the OL21 force field [55] for nucleic acid simulations, and the GAFF force field [56] for organic small molecules. Popular MD simulation programs include GROMACS, LAMMPS, AMBER, NAMD, and the Forcite module in Materials Studio. However, the accessible timescale of AA‐MD is typically limited to a few hundred nanoseconds, which constrains its ability to describe slow diffusion or long‐term degradation behavior [57].

CG‐MD simulation offers significant advantages for investigating biomolecular systems characterized by complex, hierarchical length and time scales [48, 58, 59]. In CG models, groups of fine‐grained atoms are represented as coarse‐grained sites through a mapping process that reduces system complexity while preserving essential physical properties. The interactions between CG sites are effectively parameterized to capture the key dynamics and energetics of the original system under simplified equations of motion. Compared with fully atomistic simulations, CG‐MD provides three main advantages. First, the reduced number of particles decrease the degree of freedom of the simulation system and enables the simulation of larger systems over longer time and length scales. Second, by smoothing out high‐frequency fluctuations inherent in atomistic models, CG simulations allow the use of larger integration time steps, thereby enhancing sampling efficiency. Third, the development of CG models offers implicit insights into both molecular architecture (via mapping strategies) and system energetics (via simplified interaction potentials), contributing to a deeper understanding of mesoscopic and macroscopic phenomena. These benefits make CG‐MD an invaluable tool for exploring complex biological systems beyond the reach of traditional all‐atom simulations and continue to drive its methodological development and widespread application [60]. Nevertheless, the coarse‐graining process inevitably sacrifices atomic resolution and may lead to less accurate descriptions of specific host–guest interactions or metal coordination geometry [61].

Monte Carlo (MC) method [62] typically refers to the importance sampling method in molecular simulations, which explores configurational space through stochastic rather than deterministic processes [63]. In a typical MC cycle, a trial configuration is first generated at random. The energy or relevant thermodynamic property of this configuration is then evaluated to compute a probability factor, often based on the Boltzmann distribution. This probability is subsequently compared with a randomly generated number to determine whether the trial configuration should be accepted or rejected, following predefined acceptance criteria. Through iterative sampling, the MC method enables efficient exploration of equilibrium states, especially in systems with complex energy landscapes or rare events. A notable extension is the grand canonical MC (GCMC) method, which integrates the principles of the grand canonical ensemble with MC sampling, allowing for accurate prediction of adsorption behavior, chemical potential, and other thermodynamic properties in open systems [64]. It is worth noting that MC simulations are particularly efficient for equilibrium sampling, as they explore configurational space through stochastic trial moves without integrating time‐dependent equations of motion. In contrast, MD simulation provides detailed dynamic trajectories that describe atomic motions and temporal evolution of the system. Therefore, MC and MD serve complementary purposes: MC excels at equilibrium property estimation, while MD is indispensable for analyzing kinetic and transport phenomena.

MD simulation is often combined with quantum mechanics (QM) methods to study the physical and chemical behavior of materials from a multiscale perspective. QM methods are currently the most accurate theoretical tools for analyzing the physicochemical properties of molecular systems, and are able to reveal the reaction mechanisms and energy distributions at the level of the electronic structure. However, with the expansion of the spatial scale of the system under study, the quantum effect is gradually weakened or can be ignored, and the behavior of the system becomes more consistent with the laws of classical statistical mechanics. For processes that do not involve traditional chemical bond breaking and formation (e.g., adsorption and diffusion of pollutants at interfaces), classical MD simulation has significant advantages in terms of computational efficiency and applicability. QM/MD provides unique insight into bond formation/breaking, framework hydrolysis, and pH‐dependent coordination dynamics, though its high computational cost limits its application to short timescales or small systems [65].

As early as 1927, scientists used QM to successfully describe the formation of chemical bonds between two hydrogen atoms in a hydrogen molecule, and this study is considered the starting point of quantum chemistry. The core of QM lies in the solution of the many‐body Schrödinger equation, which cannot be solved analytically at present, and a number of approximation methods have been developed to balance computational accuracy and efficiency, such as the configuration interaction method [66], multibody perturbation theory [67], density functional theory (DFT) [68], and various semi‐empirical quantum chemical methods. These methods have been widely used to study molecular mechanisms in the fields of environment, materials, life sciences, and medicine [69, 70, 71].

Among them, DFT is one of the most widely used methods in current quantum chemistry research. It is based on the Hohenberg–Kohn theorem, which expresses the ground state energy of a system as a generalized form of the electron density, thus effectively simplifying the problem of 3N degrees of freedom in the multielectronic system into an electron density function that depends on the three coordinate dimensions, and significantly reducing the computational complexity [68]. According to the theory, the electron density distribution uniquely determines the ground state energy and properties of the system. Common approximation methods include local density approximation [72], generalized gradient approximation (GGA) [73], meta‐GGA [74], and hybrid functionals [75], which show higher accuracy in many systems [76]. Since DFT can significantly reduce the computational cost while maintaining reasonable accuracy, it is widely used in practical problems such as drug design, catalyst development, and interfacial reaction modeling.

First‐principles calculations are also used to study the structure and properties of periodic materials or surface systems [77]. Strictly speaking, first‐principles computing refers to computational methods that solve the fundamental equations of QM from scratch, without relying on experimental parameters, and is therefore also called “ab initio methods.” Currently, the most common first‐principles computational frameworks are based on DFT theory, which is widely used in materials science and nanostructure design research. Typical quantum chemistry simulation software includes commercial software such as Gaussian, DMol3, CASTEP, and VASP in Materials Studio and open source/academic licensed software such as ORCA, Turbomole, CP2K, xtb, and others. These tools support the optimization of small molecule structures to the design of nanostructures. These tools also support a wide range of computational tasks from small molecule structure optimization to periodic crystal energy band structure analysis, addressing research needs at different scales and system complexities.

### 2.2 Force Fields for MOFs and Biomolecules

The accuracy and reliability of MD simulation strongly depend on the choice and parameterization of force fields, which define the potential energy surface governing atomic interactions. For MOF‐based DDSs, force field selection is particularly challenging due to the hybrid nature of the system, comprising metal nodes, organic linkers, and guest biomolecules with diverse chemical environments.

Conventional biomolecular force fields such as AMBER, CHARMM, and OPLS‐AA have been widely used to model drug molecules, peptides, and solvent species. However, these force fields are generally not transferable to metal–ligand coordination environments found in MOFs, where bond polarization, metal–oxygen covalency, and coordination flexibility play crucial roles. Consequently, specialized parameter sets such as Universal Force Field (UFF) [78], DREIDING [79], UFF4MOF [80], MOF‐FF [81], and QuickFF [82] have been developed to reproduce experimental lattice constants, mechanical stability, and adsorption behavior of MOFs.

Accurate parameterization strategies for MOF‐biomolecule systems often involve combining MOF‐specific force fields with biomolecular ones through hybrid or interface models [83]. For instance, MOF regions can be described using UFF4MOF or QuickFF, while organic guest and solvent components employ AMBER or CHARMM parameters. Cross‐interaction terms, primarily the Lennard–Jones (van der Waals) parameters, are usually obtained via Lorentz–Berthelot combination rules or refined using quantum‐mechanical calibration. Electrostatic (Coulombic) interactions, by contrast, are not mixed through combination rules but are determined solely by the assigned partial charges on each subsystem.

Despite these advances, modeling such heterogeneous systems remains nontrivial. Partial charge assignment for metal centers, dynamic coordination changes, and framework flexibility often require reparameterization or the incorporation of polarizable or reactive force fields (ReaxFF) [84]. Recent studies also report the use of ML‐derived force fields trained on ab initio datasets to achieve quantum‐level accuracy while maintaining MD efficiency [85].

### 2.3 Key Analysis Methods

The trajectory data generated from MD simulation contain rich structural and dynamical information, which can be quantitatively analyzed to elucidate the molecular mechanisms governing drug loading and release within MOF‐based DDSs. Several analytical tools have been developed to extract such insights, linking microscopic interactions with macroscopic behavior.

RDF is among the most fundamental tools used to characterize the spatial correlation between specific atom pairs, such as drug–metal centers, drug–oxygen atoms of linkers, or hydrogen‐bond donors and acceptors [86]. Peaks in the RDF indicate preferred intermolecular distances and coordination geometries, thereby revealing dominant interaction types (e.g., hydrogen bonding, π–π stacking, or electrostatics). For instance, RDF analysis can identify whether adsorption is localized near the metal nodes or distributed across organic linkers.

Hydrogen‐bond occupancy and lifetime analyses provide dynamic information on the stability and frequency of hydrogen bonds between drug molecules and the MOF framework. These metrics reflect the strength and persistence of specific interactions that govern both adsorption and release behavior. Long‐lived hydrogen bonds often indicate stronger confinement and slower release kinetics, whereas transient hydrogen‐bond dynamics correlate with faster desorption processes.

Diffusion coefficients, derived from mean‐square displacement (MSD) calculations, quantify the mobility of drug molecules inside MOF pores or across the solvent–MOF interface [87]. Variations in diffusion coefficients under different conditions (e.g., pH, temperature, or functionalization) can be directly correlated with experimental release rates, providing valuable guidance for optimizing MOF pore size and surface chemistry [88, 89].

To further understand the thermodynamic driving forces of adsorption and transport, free energy and potential of mean force (PMF) calculations are often performed [90]. PMF profiles, obtained via umbrella sampling or meta‐dynamics, describe the free energy landscape along reaction coordinates such as the drug's diffusion path through the MOF channels. The height of the energy barrier in the PMF curve directly reflects the difficulty of molecular diffusion, while local minima correspond to stable adsorption sites [91].

Collectively, these analytical methods allow MD simulation to bridge the gap between atomic‐scale interactions and experimentally measurable properties such as loading efficiency, release rate, and stability.

## 3 MD Simulation for Drug Loading and Interactions in MOFs

As discussed in the previous section, MD simulation provides a powerful framework for quantifying atomic‐scale interactions, diffusion dynamics, and energy landscapes in MOF‐based DDSs. Building upon these analytical methods, this section focuses on how MD simulation has been applied to elucidate the mechanistic principles of drug loading and the intermolecular interactions governing host–guest behavior in MOFs. These insights form the theoretical foundation for optimizing MOF design and tailoring drug–carrier compatibility.

### 3.1 Drug Adsorption Mechanisms

**FIGURE 4:**
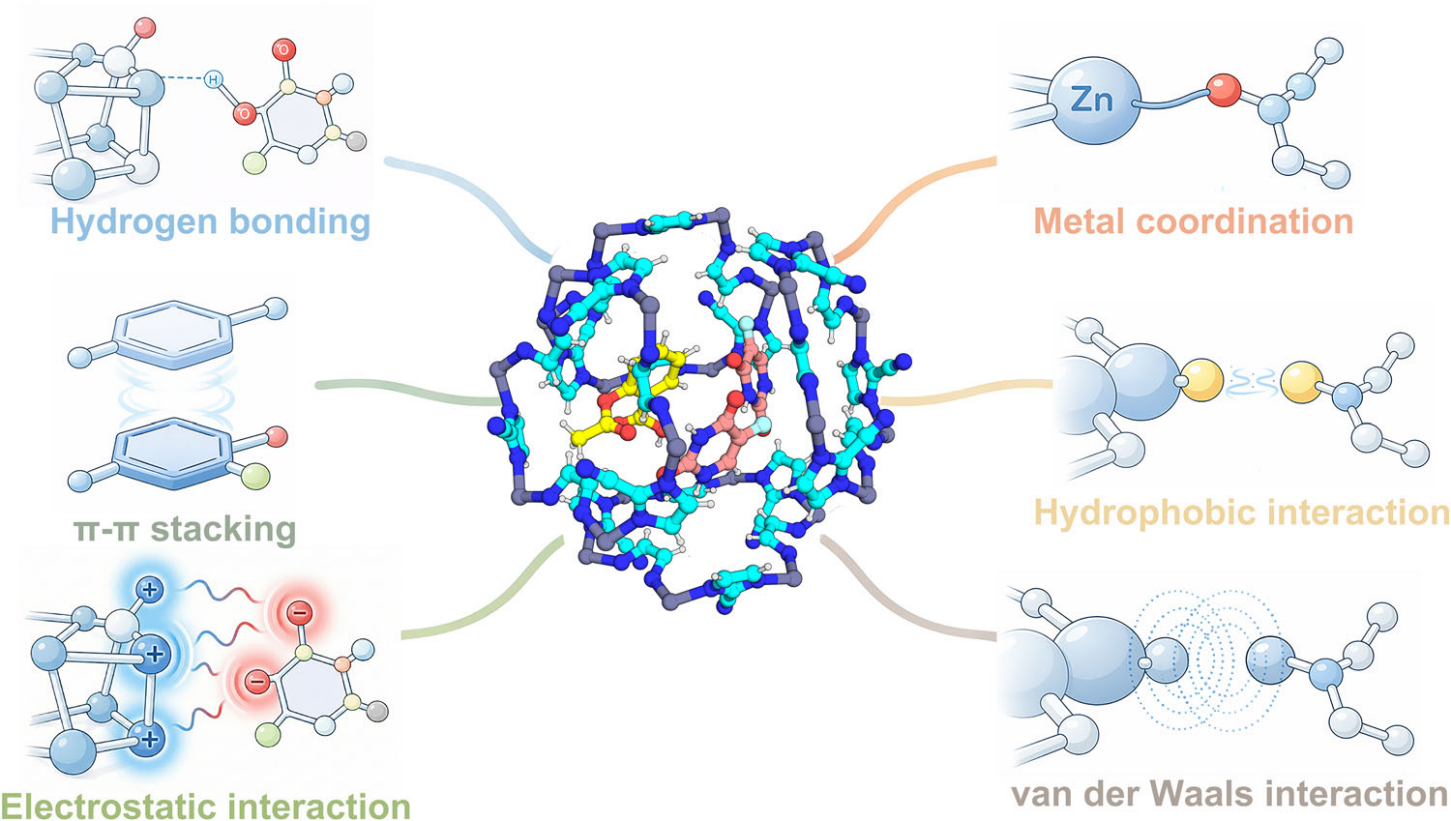
Major interaction modes involved in drug–MOF host–guest systems. The schematic illustrates hydrogen bonding, π–π stacking, electrostatic attraction, metal coordination, hydrophobic interaction, and van der Waals interaction as key forces that determine drug‐loading affinity and release behavior in MOF‐based drug delivery systems.

MD simulation has revealed that drug adsorption within MOFs is governed primarily by a combination of electrostatic, hydrogen‐bonding, π–π stacking, and van der Waals interactions [92], as Figure 4 shows. RDF and hydrogen‐bond analyses demonstrate that polar drugs preferentially interact with coordinatively unsaturated metal sites or oxygen atoms of organic linkers, while hydrophobic and aromatic drugs often anchor through π–π stacking with benzene or imidazole rings [88, 89, 93].

For example, simulations of temozolomide (TMZ), alendronate, and 5‐fluorouracil (5‐FU) in University of Oslo Framework‐66 (UiO‐66) frameworks [94] revealed that hydrogen bonds between the carboxyl or amine groups of the drug and the linker oxygen atoms significantly enhance adsorption affinity. In contrast, neutral and bulky drugs such as paclitaxel (PTX) [95] exhibit weaker interactions, relying more on physical confinement and van der Waals stabilization. MD‐derived binding free energy and energy decomposition analyses further quantify the relative contribution of these noncovalent forces, offering a molecular‐level rationale for experimentally observed loading capacities.

These results collectively indicate that the strength and selectivity of adsorption are dictated by the chemical complementarity between drug functional groups and MOF surface sites, knowledge that can directly guide the rational selection of frameworks for specific therapeutic molecules.

### 3.2 Effect of MOF Functionalization

Surface functionalization and linker modification are widely employed to tune drug–MOF interactions, and MD simulation provides atomic insight into how these chemical modifications alter adsorption thermodynamics and dynamics. The introduction of hydrophilic groups (‐NH_2_, ‐COOH, ‐OH) enhances hydrogen bonding and electrostatic interactions, resulting in stronger confinement and higher loading efficiency [96, 97, 98]. Conversely, hydrophobic modifications (e.g., ‐CH_3_, ‐CF_3_) can reduce polarity and favor sustained release by weakening guest–host attraction [99, 100].

Functionalization affects not only binding energy, but also the site and configuration of drugs within the MOF channels. For example, simulations of the NH_2_‐Institute Lavoisier Framework‐53(Fe) (NH_2_‐ MIL‐53(Fe)) system [97] show that the 5‐FU drug binds to the metal nodes of the MOF via its oxygen atoms through bonds with the organic linker. However, in the case of NH_2_‐MIL‐53(Fe), it is the nitrogen atom of the amino functional group that exhibits stronger interactions with the fluorine atom of 5‐FU. These computational findings reveal that the strategic design of linker functionalization can modulate the adsorption sites and configurations of drugs, thereby optimizing the performance of DDSs.

### 3.3 Loading of Various Therapeutic Agents

Beyond small‐molecule adsorption studies, MD simulation has been increasingly employed to investigate the encapsulation of diverse therapeutic agents in MOF‐based DDSs. The ability of MOFs to host drugs with different sizes, polarities, and functional groups provides a versatile platform for tailoring delivery performance, while MD simulation offers atomistic insight into the driving forces that govern loading efficiency and spatial distribution within the framework.

For anticancer and antibiotic drugs, MD simulation has revealed distinct adsorption behaviors that depend strongly on molecular polarity and charge distribution. In systems such as 5‐FU@MOF‐801 [101], hydrogen bonding and electrostatic interactions dominate the stabilization of the drug within the pore environment. Conversely, for hydrophobic or bulky molecules (e.g., doxepin [102] or ibuprofen (IBU) [103]), van der Waals forces and confinement effects play a more significant role, often leading to adsorption at pore entrances or defect sites rather than deep pore penetration.

In recent years, MD studies have extended to bio‐macromolecular therapeutics, including short peptides, nucleic acids, and photosensitizers [94, 104, 105, 106, 107]. The encapsulation of peptide fragments in Isoreticular Metal–Organic Framework‐54‐II (IRMOF‐74‐II) and IRMOF‐74‐III [106] demonstrated that electrostatic attraction and hydrogen‐bond networks contribute to both structural stabilization and orientation control of the biomolecule within the pore. Similarly, simulations of single‐stranded DNA and double‐stranded DNA interacting with Zr‐BTB MOF [104] indicate that hydrogen bonds and π–π interactions primarily govern adsorption. This provides a theoretical basis for the experimental loading of DNA onto MOFs.

Overall, MD simulation provides a molecular‐level understanding of how size, charge, and functional diversity of therapeutic agents influence their accommodation and orientation within MOFs.

## 4 MD Simulation for Drug Release and Stability of MOFs

Building upon the molecular understanding of drug‐loading mechanisms discussed in the previous section, MD simulation has also been extensively employed to explore drug release dynamics and structural stability of MOF‐based DDSs under physiological conditions. These studies bridge the microscopic mechanisms of host–guest interactions with experimentally measurable macroscopic behaviors such as release rate, responsiveness, and framework degradation.

To deepen the mechanistic understanding of high drug‐loading and controlled release phenomena in MOF‐based DDSs, recent MD studies have provided quantitative analyses of RDFs, hydrogen‐bond occupancy, and drug diffusion coefficients. For example, in a cisplatin@MOF study, the RDF peak heights indicated strong guest–host interactions and preferential drug localization at pore walls, correlating with enhanced loading efficiency [108]. In another doxorubicin/MOF–graphene oxide (DOX/MOF–GO) composite investigation, hydrogen‐bond occupancy and contact number analyses revealed that polymer coating significantly reduced guest–framework interactions, leading to higher self‐diffusion coefficients for the drug and faster release kinetics [109, 110]. These types of MD‐derived metrics allow a direct linkage between structural/energetic descriptors and macroscopic performance, thus bridging the gap between descriptive loading/release results and the underlying atomistic mechanism.

### 4.1 Stimuli‐Responsive Release Mechanisms

One of the most important advantages of MOFs as drug carriers is their tunable response to external stimuli such as pH, temperature, ionic strength, or redox environment. MD simulation allows visualization of how these factors modulate drug–MOF interactions in real time. For instance, in acidic media, protonation of linker groups or guest molecules can weaken hydrogen bonding and electrostatic attraction, leading to accelerated drug release. Conversely, in alkaline conditions, deprotonation often enhances Coulombic attraction between charged linkers and drug molecules, thereby retarding desorption [108].

Temperature‐dependent MD simulation further reveals that elevated thermal motion reduces the energy barrier for release [93], a phenomenon consistent with experimentally observed thermally triggered delivery [111]. These findings demonstrate how MD offers atomistic insight into stimuli‐responsive control mechanisms that govern drug liberation from MOF carriers.

### 4.2 Diffusion Kinetics of Drug Molecules

The diffusion of drugs within MOF frameworks is a key determinant of release profiles. MD simulation quantifies this process by calculating MSD and self‐diffusion coefficients (*D*). Results show that diffusion kinetics are strongly correlated with pore geometry, surface functionality, and drug–framework affinity [112, 113, 114].

Recent MD studies, such as the work by Thompson et al. [114] on cisplatin encapsulated within zeolitic imidazolate frameworks (ZIFs), have provided valuable insight into the pH‐dependent diffusion behavior of drug molecules in MOFs. Under neutral conditions, the drug exhibits relatively stable confinement within the pore network, characterized by a gradual increase in MSD over time. However, when simulated under acidic environments, protonation of imidazolate linkers weakens Zn‐N coordination bonds, leading to partial framework flexibility and enlarged pore apertures. These structural fluctuations significantly enhance the mobility of cisplatin molecules, resulting in an increased diffusion coefficient and accelerated release. The MSD profiles reported in this study reveal a sublinear time dependence, indicative of anomalous or hindered diffusion, where drug molecules undergo intermittent hopping between transient binding sites rather than continuous motion. These results emphasize how the dynamic nature of the MOF framework, modulated by environmental conditions, governs the balance between structural integrity and molecular mobility, providing a microscopic explanation for the controlled release characteristics of pH‐sensitive MOFs.

### 4.3 Hydrolytic and Physiological Stability

The hydrolytic and physiological stability of MOFs plays a decisive role in determining their suitability as drug carriers, influencing both their degradation behavior and release kinetics. MD simulation, when coupled with experimental observations, provide valuable atomistic insight into how environmental factors such as pH and solvent composition regulate the framework's integrity.

In particular, the study by Dou et al. [102] on doxepin‐loaded ZIF‐8 demonstrated that the framework exhibits distinct stability responses under different pH conditions. Under neutral and mildly basic environments, ZIF‐8 maintained its crystallinity, with only limited thermal fluctuations in Zn‐N coordination bonds. However, in acidic media, protonation of imidazolate linkers significantly weakened Zn‐N coordination, triggering partial framework distortion and enhanced flexibility. These dynamic changes facilitated increased solvent penetration and accelerated drug diffusion, explaining the experimentally observed faster release in low‐pH conditions.

Overall, these findings emphasize that MOF stability is not static but dynamically coupled to environmental stimuli. MD simulation offers a powerful approach to quantify the subtle balance between framework rigidity and flexibility, revealing how minor variations in protonation or hydration can trigger large‐scale structural transformations. Such mechanistic understanding guides the rational design of next‐generation MOFs with enhanced aqueous and physiological durability, ensuring predictable and biocompatible drug delivery performance.

## 5 MD Studies Across Different MOF Families

In current research, the use of MOFs as DDSs mainly includes several mainstream classes of materials. IRMOFs, ZIFs, and Institute Lavoisier Frameworks (MILs) are three types of highly versatile and structurally diverse materials. IRMOFs are distinguished by their tunable porosity, which is characterized by the presence of Zn_4_O_6_^+^ tetrahedra and organic ligands, resulting in a three‐dimensional porous network structure. The unique features of IRMOFs are attributable to the use of different linkers. ZIFs are grown from M(Im)_4_ tetrahedra (M: Zn^2+^ or Co^2+^, Im: imidazolate) constructed by copolymerization between an imidazolate linker and cations [115], in which imidazolate bonds are connected by N atoms and provide tunable nanoscale ZIFs [116]. Furthermore, ZIFs offer excellent thermal and chemical stability, rendering them optimal for drug storage and controlled release. MILs, on the other hand, have unique chemical and structural properties that make them suitable for a wide range of drug carriers. MOFs synthesized via the MIL approach exhibit porous structures formed by the coordination of metal ions (e.g., Fe, Al, Mn, Cr, and Ti) with organic ligands. In the field of metal‐ion‐based MIL synthesis, Fe ions have been identified as a preferred choice due to their comparatively lower toxicity to normal cells when compared with other metal cations [117]. These nanocarriers have the potential for loading and releasing various therapeutic agents, particularly anticancer drugs [118]. The UiOs and porous coordinated networks (PCNs) are considered extremely promising DDSs due to their excellent mechanical and long‐lasting chemical stability. UiO‐based MOFs have demonstrated significant potential in the fields of antimicrobial, anti‐inflammatory, and anticancer therapies [119]. Furthermore, they exhibit excellent thermal stability, chemical stability, acid‐base stability, photocatalytic activity, and biocompatibility, offering novel approaches and solutions to address current challenges in antimicrobial, anti‐inflammatory, and anticancer treatments [120, 121]. However, further research is needed to explore the potential of PCNs as DDSs during MD simulation. Several studies have shown that MOFs materials such as IRMOFs, ZIFs, MILs, and UiOs have strong potential as DDSs. Their drug‐carrying and drug‐releasing behaviors have been extensively explored and optimized in simulation studies, making them the mainstream research materials for MOFs in drug delivery applications. To provide a clearer overview of representative MOF families applied in DDSs, Table 1 summarizes their general chemical formulas, typical metal–ligand compositions, and key advantages and challenges. These MOFs differ significantly in topology, coordination geometry, and physiochemical stability, which collectively determine their suitability for drug encapsulation, diffusion, and release. For instance, IRMOFs and ZIFs exhibit highly tunable pore sizes and excellent porosity, favoring high drug‐loading efficiency, while MILs and UiOs demonstrate better biocompatibility and chemical stability in aqueous or physiological environments. Meanwhile, Cu‐based Hong Kong University of Science and Technology (HKUST) frameworks are distinguished by their redox activity and high surface sensitivity, although they tend to be less stable under acidic conditions. Overall, the characteristics summarized in Table 1 highlight the structural diversity and functional complementarity of various MOF families, laying the foundation for subsequent MD simulation studies discussed in this section.

**TABLE 1:** The major MOF classifications.

MOF	Metal ligand	Generic formula	Biocompatibility	Stimuli responsiveness	Drug delivery advantages	References
Isoreticular metal−organic frameworks (IRMOFs)	Zn	[Zn_4​_O(BDC)_3_]	Moderate; biodegradable	pH‐sensitive; thermal instability	High porosity and tunable pore size enable large drug‐loading capacity and diffusion control.	[122, 127, 128, 129, 130]
Zeolitic imidazolate frameworks (ZIFs)	Zn, Co	[M(Im)_2_]	Generally low cytotoxicity	Strong pH‐responsiveness	pH‐triggered release; endosomal escape	[131, 132, 133, 134]
Materials of Institute Lavoisier Frameworks (MILs)	Fe, Cr, V, and so on	[M(OH)(BDC)]_n_	Excellent	pH‐ and redox‐responsive	Stimuli‐responsive release; magnetic targeting	[135, 136, 137, 138, 139, 140]
University of Oslo (UiOs)	Zr	[Zr_6_​O_4_​(OH)_4_​(BDC)_6_]	Highly biocompatible; low systemic toxicity; slow degradation	pH‐ and phosphate‐responsive	Excellent stability; controllable release	[141, 142, 143, 144, 145]
Hong Kong University of Science and Technology (HKUSTs)	Cu	[Cu_3_​(BTC)_2_]	Moderate; potential ROS generation	Redox‐ and pH‐sensitive	Redox‐triggered release; photothermal therapy	[136, 146, 147, 148, 149, 150]

Abbreviations: BDC, 1,4‐benzenedicarboxylate; BTC, 1,3,5‐benzenetricarboxylat; Im, imidazolate or substituted imidazole linkers; M, metal ligands such as Zn^2+^, Co^2+^, Fe^3+^, Cr^3+^, and so on.

### 5.1 IRMOFs

In 2002, Yaghi's team conducted pioneering research on MOFs and successfully synthesized MOF‐5. This novel structure was constructed from octahedral Zn‐O‐C clusters and benzene chain structures, and later became part of a series of MOFs known as IRMOFs [122]. IRMOFs are an ideal choice for specific drug carriers due to their outstanding properties, including good biocompatibility, excellent slow drug release, and high drug‐loading capacity [123].

Kotzabasaki et al. [112] explored the potential of IRMOFs in drug storage and release by examining the interaction between IRMOF‐74‐III, its functionalized derivative OH‐IRMOF‐74‐III, and the anticancer drug gemcitabine (GEM). Their study offers theoretical support for the use of IRMOFs in DDSs. The geometries of both MOFs were optimized using DFT to ensure simulation accuracy. Subsequently, at 37°C, the adsorption properties of GEM under various pressures (10^−15^ to 10^5^ Pa) were simulated using GCMC methods. The results indicated that each gram of MOF could adsorb up to 1130 mg of GEM, corresponding to approximately 75 drug molecules per simulation cell. In terms of drug loading, both IRMOF‐74‐III and OH‐IRMOF‐74‐III achieved capacities of 400 mg GEM/g [124], outperforming many other carriers and approaching that of liposomes (1500 mg GEM/g) [125], highlighting their promise for GEM storage. MD simulation was conducted to investigate GEM diffusion in the two MOFs. At low loading (32 wt%), GEM showed higher diffusion coefficients in OH‐IRMOF‐74‐III, attributed to the hydroxyl groups providing auxiliary interactions that facilitate dispersion. At high loading (95 wt%), the diffusion coefficients became comparable. Overall, both IRMOF‐74‐III and OH‐IRMOF‐74‐III exhibit high loading capacities and favorable diffusion properties, making them promising candidates for efficient GEM delivery and controlled release in drug delivery applications.

Liu et al. [126] have extended understanding of the potential of IRMOFs as drug carriers through an extensive study. They used GCMC simulation to evaluate the adsorption properties of 28 different MOFs for the antihypertensive drug amlodipine. The study identified 19 MOFs as promising carriers for amlodipine, with the help of MD simulation. Five deformations in the IRMOF‐74 series (IRMOF‐74‐V‐hex, IRMOF‐74‐IV, IRMOF‐74‐VII‐oeg, IRMOF‐74‐VII, and IRMOF‐74‐IX) exhibit exceptional properties. They not only have relatively high drug adsorption but also perform well in terms of controlled‐release properties and biocompatibility. This suggests that these IRMOFs can serve excellently as drug carriers for amlodipine.

And a study conducted by Khedri et al. [151] in 2021 evaluated the adsorption properties of the drug phenanthridine on three MOFs: IRMOF‐16, HKUST‐1, and ZIF‐8 using MD simulation. These MOFs were selected for their excellent performance in π–π stacking, hydrogen bond formation, and electrostatic interactions. To further analyze the adsorption mechanism of PHP on these MOFs, the research team also employed CG simulation and DFT calculation. The simulation results successfully illustrate the progressive nature of the drug adsorption process over the complete 100 ns simulation and that IRMOF‐16 has the highest adsorption efficiency of 100% for PHP among all MOFs. HKUST‐1 and ZIF‐8 have slightly lower relative adsorption efficiencies of 60 and 40%, respectively. The adsorption capacity of IRMOF‐16 for PHP is validated by both CG simulations and DFT calculations, with adsorption energies of −343.4 kJ/mol and −1.53 eV, respectively. They attempted to enhance the PHP adsorption capacity of IRMOF‐16 through three different modification methods: hydroxyl modification, pore size adjustment, and formation of a hybrid framework by creating a covalent–organic frameworks (COFs) with DAAQ‐TFP. The efficiency of PHP adsorption by IRMOF‐16 can be enhanced by introducing hydroxyl functional groups and binding with COFs. This modification increases the negative net surface charge, resulting in a stronger interaction between the positively charged PHP molecules and the material surface. However, enlarging the pore size of IRMOF‐16 may reduce its adsorption performance by limiting the interaction of PHP molecules with the active sites. This work demonstrates the potential for gaining insight into the adsorption mechanisms of drug carrier materials through MD simulation. It also suggests that material modifications can enhance the functionality of MOFs in DDSs. IRMOFs are a new category of materials with high porosity that show great potential in the field of drug delivery. Through chemical and functional group modification, IRMOFs can enhance drug loading and control its release, opening up new possibilities for more efficient DDSs. Although still in the exploration and improvement stage, IRMOFs have become an important focus for future drug delivery research and applications.

The studies on IRMOF‐based systems consistently demonstrate their high porosity and structural tunability, which make them attractive hosts for a wide range of drug molecules. Nevertheless, MD simulation also reveals important challenges: the behavior of Zn‐O‐C coordination environments is not always captured accurately by existing force fields, and hydrolytic degradation, known to occur experimentally under physiological conditions, cannot be fully explored within nanosecond‐scale simulations [152, 153]. These limitations highlight the need for improved parameterization strategies and longer‐timescale modeling. At the same time, the atomistic insights obtained from IRMOF simulations, such as drug–linker interaction energies and pore‐occupancy profiles, suggest promising pathways for rational design. Adjusting linker chemistry, modifying pore apertures, or introducing functional substituents are all strategies that simulations indicate could improve drug loading, guide‐controlled release, and enhance compatibility with biological environments. A closer integration of MD predictions with experimental measurements will likely accelerate the development of IRMOF‐based DDSs optimized for real therapeutic conditions.

In summary, MD studies on IRMOFs have revealed their high porosity and tunable pore chemistry as key determinants of drug adsorption and diffusion. The interaction strength between functionalized linkers and drug molecules largely governs loading efficiency, while the rigidity of the Zn‐O cluster framework ensures structural stability during release. Despite their relatively simple architecture, IRMOFs serve as ideal model systems for understanding fundamental host–guest interactions in MOF‐based DDSs, providing a baseline for comparison with more complex frameworks such as MILs and UiOs. These insights establish IRMOFs as benchmark references for evaluating the performance and mechanism of other MOF families.

### 5.2 ZIFs

ZIFs are materials composed of transition metal ions, such as zinc or cobalt, and imidazoline‐based ligands with linkers formed in a tetrahedral geometry [131]. The nitrogen atoms of the imidazole ring act as bridges connecting the metal centers in a three‐dimensional framework. The crystal structure of ZIFs is similar to that of conventional zeolitic materials, with comparable topological features [154]. These materials have a porous structure and can hold a high substance load. They are sensitive to degradation under different pH conditions but exhibit good thermal and chemical stability [155].

Kulkarni et al. [156] conducted MD simulation to investigate the molecular‐level interaction between titanocene dichloride and lactoferrin (Lf) while studying MOFs of ZIF‐8 nanoparticles encapsulating the core–shell structure of protein‐titanium complexes. The study identified glutamic acid, histidine, and aspartic acid as the specific amino acids that undergo major interactions with the titanium cation. In addition to the amino acids, a few tyrosine residues also bind to the titanium cation. The MD simulation trajectories show that these residues form stable bonds with the titanium cation. During the simulation, the protein bound to titanium exhibited a root‐mean‐square deviation (RMSD) value of less than 2 Å relative to its initial structure throughout the simulation. The final stage of the simulation indicated the formation of a stable binding state between Lf and titanium ions. MD simulation confirmed the conjugation of titanocene dichloride with Lf, while they confirmed the formation of Lf/titanocene dichloride and loaded ZIF‐8 NPs using Fourier transform infrared spectrometer, powder X‐ray diffractometer, Raman spectroscopy, and Ultraviolet–near‐infrared (UV–NIR) spectroscopy. Therefore, the ZIF‐8 framework has the potential to be used as a nanoplatform for tumor phototherapy.

Sun et al. [157] investigated the adsorption behavior of four antiepileptic drugs: gabapentin (GBP), levetiracetam (LEV), phenytoin sodium (PHT), and valproate (VPA), in ZIF‐8, ZIF‐67, and ZIF‐90 using MD simulation. The results showed that in ZIF‐8, drug adsorption decreased with increasing molecular volume and ring number. GBP and VPA exhibited similar adsorption capacities per unit cell, likely due to GBP's carboxylic acid and amide groups compensating for its smaller ring structure. The simulated weight loadings of VPA, GBP, LEV, and PHT in ZIF‐8 were 8.5, 9.94, 9.52, and 12.01%, respectively, which agreed well with experimental results (6–14 wt%) obtained via Ultraviolet–Visible (UV–vis) spectrophotometry. Due to the significantly lower adsorption observed in ZIF‐67 and ZIF‐90, further analyses focused on ZIF‐8. Concentration profiles indicated that GBP accumulated more in the inner region of ZIF‐8, reflecting stronger interactions with the framework, whereas PHT showed the lowest central concentration. MSD analysis yielded self‐diffusion coefficients of 1.04 × 10^−4^, 1.07 × 10^−4^, 9.13 × 10^−5^, and 1.03 × 10^−4^ cm^2^ s^−1^ for VPA, GBP, LEV, and PHT, respectively, supporting the suitability of ZIF‐8 for controlled drug delivery. Given its high loading capacity, favorable diffusion behavior, and slow degradation in vivo, ZIF‐8 was identified as an effective nanocarrier for specific antiepileptic drugs.

Dou et al. [102] investigated the use of ZIF‐8 nanoparticles for delivering the antidepressant drug doxepin, employing the Gibbs–MC method to evaluate its adsorption and release properties. The ZIF‐8S model, composed of microcrystals or nanocrystals, was found to retain high porosity under experimental conditions. Adsorption isotherms indicated that ZIF‐8 adsorbed doxepin at a rate of 8.6%, which increased to 12% within a fixed‐diameter pore cavity. However, in the presence of solvent, the adsorption efficiency dropped to 2.1%, as ethanol molecules occupied up to 32% of the available adsorption sites. This reduction was attributed to the small size, hydroxyl groups, and hydrogen‐bonding ability of ethanol, which allowed it to dominate the pore space. Energy distribution analysis and isothermal heat calculations further confirmed that ethanol adsorbs more readily than doxepin under identical conditions, requiring less energy to occupy adsorption sites. Comparison of the energy distributions showed that doxepin adsorption is more favorable in the absence of solvent. MD simulation revealed that the self‐diffusion coefficient of doxepin in ZIF‐8 increased from 7.353 × 10^−6^ to 1.004 × 10^−4^ cm^2^ s^−1^ upon solvent removal, suggesting that eliminating solvent enhances both drug loading and controlled release. Overall, the study supports ZIF‐8 as an effective carrier for doxepin delivery but emphasizes the need for optimizing MOF selection and solvent systems to further improve storage and release performance in biomedical applications.

Gomar et al. [158] systematically investigated the adsorption properties of two anticancer drugs, 5‐FU and thioguanine, on ZIF‐1, ZIF‐3, and ZIF‐6 using GCMC and MD simulation. Under identical conditions, ZIF‐1, ZIF‐3, and ZIF‐6 adsorbed 1.292, 3.342, and 6.124 mmol g^−1^ of 5‐FU, and 1.294, 2.699, and 4.237 mmol g^−1^ of thioguanine, respectively, with ZIF‐6 exhibiting the highest capacity due to its larger pore size. Adsorption of both drugs increased rapidly with pressure and saturated below 1 bar, suggesting that adsorption is influenced not only by pore volume but also by isosteric heat. The calculated heats of adsorption showed that drug–ZIF interactions were strongest in ZIF‐6. For 5‐FU, the heat of adsorption ranged from 84.014 to 114.913 kJ mol^−1^ in ZIF‐1, 103.566 to 140.030 kJ mol^−1^ in ZIF‐3, and 88.311 to 175.912 kJ mol^−1^ in ZIF‐6. For thioguanine, the corresponding ranges were 120.113–136.460, 104.045–159.330, and 126.986–189.521 kJ mol^−1^. The study also revealed a preference for drug molecules to adsorb in cage II (4‐membered Zn‐limidazolate ring) in ZIF‐1 and ZIF‐6. RDF analysis highlighted the important role of metal centers in drug binding. These results provide a theoretical basis for understanding drug–MOF interactions and designing more effective ZIF‐based DDSs.

Zhang et al. [159] developed a method to enhance antibody‐mediated cellular targeting by immobilizing antibodies onto nanoparticles using ZIF‐8, providing a platform for oriented antibody attachment essential for effective antigen recognition. Using MD simulation, they examined the interaction between the Fc region of an IgG antibody and a Zn‐based ZIF‐8 complex. The ZIF‐8 complex was initially positioned at six noncontact locations around an Fc dimer, and 10 ns vacuum simulations identified three preferential binding sites (BP1, BP2, BP3). These were further evaluated in aqueous solution (TIP3 water with 0.15 mol NaCl) via 20 ns unbiased MD simulation. BP2 demonstrated the highest binding energy and stable contact with ZIF‐8, whereas BP1 and BP3 lost contact during simulation, likely due to the absence of histidine residues critical for Zn coordination. Contact analysis over 20 ns confirmed that ZIF‐8 consistently interacted with BP2 but not with the other two sites. These findings highlight the strong binding affinity between ZIF‐8 and histidine residues in the Fc region, supporting the use of ZIF‐8 for antibody immobilization. This approach offers a promising and generalizable platform for targeted bio‐nanotechnology applications.

Thompson et al. [114] investigated the use of ZIFs to enhance the delivery efficiency of cisplatin in tumor therapy while minimizing its release in healthy tissues. To achieve high drug loading and an elevated energy barrier for cisplatin release, ZIFs with good biocompatibility and pH sensitivity were evaluated. An ideal carrier should enable stable encapsulation in neutral or slightly alkaline environments and promote drug release under acidic conditions typical of tumor sites. Since drug loading is determined by pore volume accessible to cisplatin, increasing the energy barrier by reducing pore size may hinder encapsulation efficiency. Alternatively, larger pores with enhanced MOF–drug interaction energies, such as via the incorporation of polar groups, can achieve high energy barriers without sacrificing loading capacity. Among the ZIFs studied, ZIF‐11, ZIF‐70, and ZIF‐82 demonstrated good cisplatin adsorption. However, ZIF‐11's small pore window limits diffusion, reducing both release rate and encapsulation efficiency. ZIF‐70, with fewer polar groups, exhibited weaker interactions with cisplatin, negatively impacting drug retention. In contrast, ZIF‐82 showed the most promising performance, combining high loading capacity with improved cisplatin immobilization through polar group modification, effectively preventing premature release. This study highlights the importance of optimizing pore size, window diameter, and functional group composition in designing ZIF‐based carriers for efficient and targeted cisplatin delivery in cancer therapy.

Ahmadzadi et al. [113] conducted a study on the encapsulation of cisplatin in ZIF. The study utilized MD simulation to investigate the encapsulation behavior of cisplatin in ZIF‐7, ZIF‐8, and ZIF‐9 at varying temperatures. The study results indicate that the system temperature significantly affects the encapsulation process. Specifically, increasing the temperature from 250 to 350 K led to a subsequent increase in the adsorption capacity of cisplatin in ZIF. When comparing the three MOFs, ZIF‐7, ZIF‐8, and ZIF‐9, it was observed that cisplatin encapsulation in ZIF‐8 was more significant. This suggests that the pore structure and chemical properties of ZIF‐8 may have a higher affinity for cisplatin, providing better conditions for adsorption and encapsulation.

In the study by Dahri et al. [160], they aimed to find materials that could inactivate the spike protein (S protein) of severe acute respiratory syndrome coronavirus 2 (SARS‐CoV‐2), thereby preventing the virus from entering and infecting human cells. The study employed three different MOFs, namely, ZIF, UiOs, and IRMOF, to induce structural changes in the S‐protein and prevent its interaction with the angiotensin‐converting enzyme 2 (ACE2) receptor on the surface of human cells. MD and molecular docking simulations were employed to assess the structural impact of MOF binding on the S protein. Secondary structure changes were analyzed using the Define Secondary Structure of Proteins tool, alongside other metrics such as S protein–MOF interaction energies, solvent‐accessible surface area, and the number of hydrogen bonds formed between the S protein and ACE2. The results indicated that a reduction in structured elements like β‐sheets and α‐helices, and an increase in disordered structures such as coils and turns, corresponds to decreased protein stability and impaired receptor interaction. While all three MOFs induced deformation in the S protein, ZIF had the most pronounced effect. Specifically, ZIF exposure led to a 21% reduction in β‐sheet content and the complete loss of α‐helices, along with an increase in disordered secondary structures. ZIF also exhibited the strongest interaction energy (IE) and formed the most hydrogen bonds with the S protein, suggesting a higher capacity to disrupt its function. These findings suggest that ZIF is a promising candidate for blocking the SARS‐CoV‐2 S protein from binding to ACE2, offering potential for the development of novel antiviral materials.

Dahri et al. [105] conducted research on a smart DDS for cancer therapy that responds to tumor‐associated enzymes, particularly Cathepsin B (CTSB), which is highly expressed in the tumor environment. CTSB is a lysosomal enzyme that plays a crucial role in the proliferation, invasion, and metastasis of cancer cells, making it an ideal target [161]. To achieve this goal, researchers developed two DOX conjugates that are bound to peptides, Fmoc–Phe–Lys–Gly–DOX and acetyl–Phe–Lys–Gly–DOX. These drug conjugates can be cleaved by CTSB and release the active drug DOX, which exerts selective killing of tumor cells [162]. To investigate the loading of drug conjugates within different MOFs, including ZIF, UiO‐66 and HKUST‐1, they used MD simulation to simulate the effect of MOFs with different structures as drug carriers and the distribution and interaction of DOX prodrugs within them under microfluidic conditions. The simulation results indicate that the stability of the drug interaction with MOFs and the formation of drug clusters are affected by the concentration of DOX prodrug. The interaction of DOX with ZIF is the most stable and effective. This information is valuable for understanding drug loading and release under microfluidic conditions. The stability of the interaction between the DOX prodrug and ZIF was higher at lower concentrations. This suggests that using lower drug concentrations in practical applications could improve therapeutic efficiency and reduce potential side effects.

Hasanzade et al. [163] investigated the adsorption and release of the anticancer drug DOX on two biocompatible ZIFs, ZIF‐7 and ZIF‐8 by MD simulation. The results showed that ZIF‐7 had a stronger binding affinity with DOX compared with ZIF‐8. This was attributed to the ability of ZIF‐7 to form stronger bonds with DOX. The low diffusion coefficients of DOX in both ZIFs suggest that these materials are suitable for controlled release of DOX, providing the possibility of slow‐release delivery of anticancer drugs. This controlled release property is crucial for reducing the side effects of chemotherapeutic drugs and improving their therapeutic efficacy.

ZIF‐based MOFs continue to attract significant attention due to their intrinsic pH‐responsiveness and structural flexibility, features that underpin many of their drug‐release behaviors. MD simulation has provided valuable insight into protonation‐mediated changes in pore accessibility and diffusion; however, accurately capturing ZIF “breathing” remains a methodological difficulty, as conventional fixed‐topology force fields often underestimate dynamic aperture fluctuations [81, 164]. These issues suggest that further improvements in flexible MOF force fields or hybrid simulation approaches will be necessary. Despite these challenges, ZIF systems benefit greatly from MD‐guided analysis, which highlights how linker protonation states, mixed‐linker compositions, and surface functionalization can be tuned to achieve pH‐triggered or site‐specific release profiles. These findings point toward a future in which simulation‐informed ZIF engineering can more precisely match drug physicochemical properties with desired therapeutic outcomes.

Overall, ZIF‐based simulations highlight the unique pH‐responsive behavior and structural flexibility of these imidazolate frameworks. MD trajectories consistently demonstrate that protonation of imidazolate linkers under acidic conditions triggers partial pore expansion, facilitating controlled drug release. Hydrogen‐bond occupancy and radial distribution analyses further elucidate how framework flexibility mediates dynamic drug–MOF interactions. These findings establish ZIFs, particularly ZIF‐8, as benchmark materials for responsive DDSs design, linking microscopic coordination dynamics with macroscopic release performance. Their dynamic adaptability under physiological stimuli represents a key bridge between structural rigidity and environmental responsiveness in MOF‐based DDSs.

### 5.3 MILs

MILs are a class of self‐assembled porous hybrid materials composed of inorganic metal ions and organic ligands [135]. The most common metal centers found in MILs are Al, Fe, Cr, and Ti. The organic ligands are primarily benzene‐1,3,5‐tricarboxylic acid (BTC), 1,4‐benzenedicarboxylic acid (BDC) or their derivatives, and fumaric acid. The first MIL‐type MOF reported in 2002 was MIL‐53(Cr), a prototypical example of a flexible MOF [165]. Since then, a number of other materials, including MIL‐88 [166, 167], MIL‐100 [168, 169], MIL‐101 [170, 171], MIL‐125 [172], and MIL‐127 [173], have also been gradually synthesized and reported.

To investigate the molecular mechanism behind MIL‐100(Fe)’s favorable adsorption of therapeutic drugs, Mileo et al. [174] developed a microscopic model and compared theoretical predictions with experimental data. The model drugs used in the experiments were caffeine, IBU, nicotine (NIC), 5‐FU, and DOX. The results indicate that the theoretical adsorption was higher than the experimental values when the drugs were allowed unrestricted access to the large cages of MOF. However, when drug access to the smaller pores of MIL‐100(Fe) was restricted [175], the theoretical predictions and experimental values were much closer. This suggests that Mileo's restriction model accurately simulates drug adsorption behavior in MIL‐100(Fe). The study analyzed the adsorption of various drugs and found that DOX had a very favorable adsorption isotherm, saturating at relatively low pressures, whereas the adsorption of 5‐FU and NIC did not behave in the same way. Although the predicted adsorption of 5‐FU in MIL‐100(Fe) was higher than in other experiments [176], researchers were concerned about the drug's ability to be released effectively in a controlled manner due to its small enthalpy of adsorption [177]. Thus, they investigated the interaction of DOX in MIL‐100(Fe). MD simulation showed that DOX molecules mainly appeared in dimeric form in the pores of the MOF. And stacking occurred very infrequently, indicating that the interaction of DOX with MIL‐100(Fe) was weak. Upon analyzing the RDF, they discovered that the hydrophobic center of the DOX molecule can interact with the organic ligands in the framework through π‐π interaction. Although this interaction is not ideal, MIL‐100(Fe), as a material with both hydrophilic and hydrophobic centers, provides a potential MOF selection that aligns with drug properties.

Tohidi et al. [138] predicted the drug loading of MIL‐100(Fe) and MIL‐100(Fe)/chitosan (CS) using MD simulation. The simulation results indicate that the drug loading of MIL‐100(Fe)/CS was 60%, compared with 26.41% for MIL‐100(Fe) without CS encapsulation. This suggests that CS encapsulation increased the drug loading by 32%. Subsequent experiments confirmed that the introduction of CS increased the drug loading to 56%. The analysis of the MSD curves of MIL‐100(Fe) particles after surface coating with CS revealed that the addition of CS effectively improved the diffusion coefficient, particle stability and drug loading. These findings suggest that introducing CS is an effective method for increasing the drug loading of MIL‐100(Fe). However, their subsequent experimental data showed that the drug loading of MIL‐100(Fe)/CS was only 47%, slightly lower than the simulation results. This discrepancy may be due to the presence of tiny pores in the experiment that are inaccessible to drug molecules. Both theoretical studies and experiments agree that the introduction of CS can significantly improve the drug‐loading capacity of the particles.

Salahshoori et al. [178] investigated the use of MIL‐53(Al) as an adsorbent for removing pharmaceutical pollutants, specifically diclofenac, ketoprofen, indomethacin, and mefenamic acid, from wastewater. The study employed QM calculation, MC simulation, and MD simulation to gain insights into the adsorption behavior of the pollutants on MIL‐53(Al). This simulation approach elucidates the interaction mechanism between drug molecules and adsorbents at the microscopic level. The study analyzed the interaction between drug molecules and MIL‐53(Al) using various techniques, including electrostatic potentials, reduced density gradients, and Hirshfeld surfaces. The results showed that drug molecules interact with the pore structure of the MIL‐53(Al) surface through van der Waals forces, hydrogen bonding, π–π stacking, and electrostatic interactions. These interactions create stable complexes between the solid surface and the organic molecules. They validated the accuracy of the model by synthesizing MIL‐53(Al) samples and using them in adsorption experiments of pharmaceutical pollutants. The agreement between the experimental data and the simulation results verified the practicality and reliability of the proposed model, indicating that MIL‐53(Al) is an effective adsorbent for pharmaceutical pollutants. This study confirms the potential application of MIL‐53(Al) in treating drug contaminants in wastewater. It also promotes the theoretical study of MILs as DDSs, which can aid in developing new application strategies in the fields of environmental treatment and pharmaceuticals.

Salahshoori et al. [179] also investigated MIL‐101(Cr) functionalized with biodegradable β‐cyclodextrin (β‐CD), as potential nanocomposite adsorbents for the removal of pollutants in water, including ketorolac (KTRK), naproxen, and tramadol. This study combined MD simulation and DFT calculations to investigate the interactions between contaminants and adsorbents. To further evaluate the adsorption capacity of MIL‐101(Cr) on the target pharmaceutical pollutants, the researchers also used MD and MC simulations. The results showed that the functionalized nanocomposite adsorbent exhibited a higher adsorption energy of −1934 kcal/mol for KTRK compared with the adsorption energy of pristine MIL‐101(Cr) (−1916 kcal/mol), which demonstrated superior adsorption performance. Although the main application directions of this study are wastewater treatment and water resource protection, the results also provide valuable references for the development of DDSs, especially for potential applications in improving drug delivery efficiency and stability.

Farzi et al. [97] investigated the interaction between the NH_2_‐functionalized MIL‐53(Fe) (NH_2_‐MIL‐53(Fe)) MOF and the chemotherapeutic drug, 5‐FU. This was achieved using a variety of methods, including classical MD, steered MD (SMD), molecular docking, and quantum theory of atoms in molecule. The results demonstrated that the 5‐FU drug was encapsulated within the channels of the MOF, and that the density of the system decreased with increasing temperature in the presence of nanoparticles. The MSD, total self‐diffusion coefficient, and the diffusion coefficient along the pore direction of the drug gradually increased with increasing temperature over time, indicating that the diffusion properties of the drug in the MOF were enhanced with temperature. Further analyses indicated strong interactions between the nitrogen atoms in the amino functional group in NH_2_‐MIL‐53(Fe) and the fluorine atoms in 5‐FU, suggesting that the amino functional group has an important influence on the adsorption of 5‐FU. Using SMD calculations, the researchers also calculated the free energy and entropy of encapsulation and release of 5‐FU into and from the NH_2_‐MIL‐53(Fe) channel. The results indicated that the release of 5‐FU from the NH_2_‐MIL‐53(Fe) channel was a spontaneous process with a free energy of −11.12 kcal/mol. These findings provide significant theoretical support for the design of metal–organic backbone‐based DDSs.

MIL‐type MOFs exhibit distinctive redox and pH‐responsive behavior, making them well suited for tumor‐microenvironment‐specific drug delivery. MD studies have shed light on how guest molecules interact within the flexible MIL framework and how coordination changes may influence release kinetics. Yet, important gaps remain: redox transitions between Fe^3+^ and Fe^2+^, key to MIL degradation mechanisms, are not captured by classical MD, and the influence of acidic or reductive physiological conditions can only be approximated indirectly [180]. Even so, the simulation results offer meaningful design insights. Adjusting linker flexibility, fine‐tuning metal–ligand coordination geometry, or incorporating redox‐reactive components may allow MIL frameworks to better exploit pathological microenvironments for controlled or sequential release. Continued efforts in combining MD with higher‐level quantum or reactive models may ultimately provide a more complete picture of MIL behavior under biological conditions.

In conclusion, MIL‐type frameworks exhibit diverse coordination environments and variable hydrophilicity, which can be finely tuned through metal node selection and linker modification. MD simulation reveals that the presence of open metal sites promotes strong electrostatic and coordination interactions with polar drugs, while surface functionalization enhances biocompatibility and stability. The combination of large pore volume and chemical tunability makes MILs highly adaptable carriers, and simulation‐guided design provides valuable insight into optimizing these materials for sustained and targeted drug delivery. Compared with IRMOFs and ZIFs, MIL frameworks demonstrate the importance of metal–ligand coordination chemistry in tailoring host–guest interactions and release kinetics.

### 5.4 UiOs

The University of Oslo has successfully synthesized a series of MOFs with Zr as the metal center [141]. These materials are known as UiOs due to their highly porous structure [20]. Cavka et al. [141, 181] were able to synthesize several different MOFs by reacting Zr^4+^ with various dicarboxylic acid ligands, including UiO‐66, UiO‐67, and UiO‐68. These MOFs are named after the ligand structures they employ. UiO‐66 is often used as a carrier for anticancer drugs due to its exceptional properties.

Li et al. [182] investigated the mechanism of drug adsorption by UiO‐66 as a drug carrier using MD simulation. However, the adsorption efficiency for IBU and 5‐FU was lower, at 20 and 30%, respectively. The results showed that UiO‐66 has high adsorption efficiency for busulfan (up to 80%) due to its chain structure and anionic groups, such as sulfonic acid groups. The study found that the interaction energies of the three drugs had a linear relationship with the ratio of the number of contact atoms. This suggests that the interaction energies of drugs in UiO‐66 can be predicted by the number of contact atoms. The study provides a theoretical basis for screening suitable drugs for loading onto UiOs.

Boroushaki et al. [94] conducted MD simulation to investigate the loading behavior of the anticancer drugs TMZ, alendronate sodium (Ald), and 5‐FU in UiO‐66 nanocarriers. The study focused on the IE between these drugs and UiO‐66, the distribution of the drugs in UiO‐66, and the mobility of the drugs at different concentrations. The simulation results show that the loading of the UiO‐66 carrier is higher when a high concentration of drug is used compared with a low concentration of drug. The distance between the drug and the UiO‐66 center also varies for different concentration systems, with the distance being farther for lower drug concentrations and closer for higher drug concentrations. This indicates that the drug molecules are more likely to be closer to the UiO‐66 center at higher concentrations. The study also analyzed the distribution of each drug in UiO‐66. TMZ was found to adsorb more toward the center of UiO‐66 and exhibited stronger interaction energies compared with the other drugs. This suggests that the lower mobility of TMZ resulted in a firmer anchoring to the interior of UiO‐66. Additionally, Ald was loaded in the internal metal centers, while 5‐FU was distributed in the metal centers on the surface of UiO‐66. These findings offer a significant theoretical foundation for designing and optimizing UiO materials as drug carriers in DDSs.

A study by Santos et al. [183] investigated the anti‐inflammatory and neuroprotective activities of a Zr‐based DDS, magnolol (Mag), and its binding to a UiO‐66(Zr) carrier (referred to as Mag@UiO‐66(Zr)). This study focused specifically on the inhibitory effect of Mag on β‐amyloid (Aβ) secretase and on the inhibition of neurotoxicity induced by AlCl_3_. As Mag showed suitable inhibitory activity in in vitro experiments [184], they further investigated its ability to inhibit 11 proteases known to influence the course of Alzheimer's disease (AD) by in silico binding studies. The study investigated the binding and dynamic stability of Mag to specific proteins through molecular docking and MD simulation. CDK2, CDK5, MARK, and PDE3B were found to have favorable binding energies to Mag and formed dynamically stable complexes in the simulations. To assess the stability of the Mag‐binding complexes, parameters such as RMSD, root‐mean‐square fluctuations (RMSF), and radius of gyration (Rg) were calculated. The simulation of the protease–Mag complex over 50 ns demonstrated a stable dynamic equilibrium. In particularly, the MARK–Mag complex exhibited the lowest average RMSD value. The data obtained from the Rg analysis indicate that the CDK2–Mag complex is the most structurally compact and stable, which may facilitate the tight binding of Mag to proteases. Their analysis of the IE of the protease–Mag complexes revealed that Mag forms thermodynamically stable complexes with specific proteases, resulting in a negative protein–Mag IE. In the study of the protective effect of Mag against AlCl_3_ neurotoxicity, it was found that the groups treated with Mag@UiO‐66 showed a dose‐dependent prevention of neutrophil infiltration. This is consistent with previous results on relative bioavailability and tissue distribution, which suggest that Mag, when impregnated with UiO‐66(Zr), can be effectively delivered to neurons across the blood–brain barrier and prevent neutrophil infiltration and neuroinflammation. In summary, the use of Mag loaded with UiO‐66(Zr) may offer improved in vivo enzyme inhibition. Additionally, it has demonstrated strong neuroprotective activity by reducing neutrophil infiltration and neuronal apoptosis. These findings highlight the potential and efficiency of UiO‐66 for drug delivery in challenging scenarios.

The study by Yuanlei et al. [185] use MD simulation to investigate the effect of 2D‐MOFs on Aβ aggregation and formation, which is a very important line of research for the treatment of AD. This is a crucial research area for AD treatment, as abnormal accumulation and aggregation of amyloid proteins are believed to be major contributors to neurodegenerative damage [186]. They used MD simulation to evaluate the interaction effects of different MOFs with Aβ proteins. The study included four different nanoparticles: UiO‐66, IRMOF‐16, HKUST‐1, and ZIF‐8. The results indicated that all of the nanoparticles affected the fibrillization process of Aβ protein to some extent, which is a key step in the aggregation of Aβ protein into amyloid plaques. It is worth noting that UiO‐66 exhibited the strongest inhibitory effect, as evidenced by its high absolute energy value, low level of contact with Aβ particles, high number of hydrogen bonds between Aβ molecules and water molecules in the presence of UiO‐66, increased instability of Aβ particles, and significant decrease in the densification of Aβ proteins. These results suggest a strong intermolecular linkage between UiO‐66 and Aβ proteins, which could effectively block or slow down the formation and aggregation process of Aβ. Taken together, MOFs, particularly UiO‐66, have the potential to block Aβ protein formation and may contribute significantly to the treatment of AD. In addition, MOFs are highly tunable, which means their chemical properties can be modified and optimized to enhance their efficacy in blocking Aβ formation.

UiO‐type frameworks, known for their exceptional stability and biocompatibility, have been widely simulated for drug delivery applications. MD studies consistently highlight the importance of hydrogen‐bonding networks and functional‐group chemistry in governing drug loading and sustained release. However, a notable gap persists: experimentally observed defect structures, such as missing linkers and cluster vacancies, are rarely incorporated into simulation models, despite their strong influence on pore accessibility and degradation profiles [187]. Incorporating realistic defect populations represents an important direction for future computational work. Nonetheless, existing MD studies already point to practical design strategies, including modifying linker substituents to control drug affinity, tuning functional‐group density to adjust hydration, and leveraging defect engineering to balance stability with biodegradability. These mechanistic insights provide a strong foundation for developing UiO‐based DDSs tailored to physiologically relevant environments.

To summarize, UiO‐type MOFs, represented by Zr‐based UiO‐66 and its derivatives, demonstrate exceptional hydrolytic and mechanical stability, making them promising candidates for in vivo drug delivery. MD studies confirm their robustness under physiological conditions and reveal how linker functionalization and defect engineering influence drug adsorption and diffusion. The balance between rigidity and accessible porosity is critical for achieving both high loading and controlled release, and future simulation work may focus on capturing defect‐induced flexibility to refine the predictive design of UiO‐based DDSs. These features position UiOs as prototypes for biocompatible and stable MOF carriers in physiological environments.

### 5.5 Other Emerging MOFs

Zhou et al. [188] investigated the physicochemical properties and bioactivities exhibited by CD‐based MOFs loaded with curcumin (Cur), denoted as Cur–CD–MOFs, in comparison with micronized Cur prepared by conventional jet milling method. The study aimed to explore novel DDSs that enhance the dissolution rate and bioavailability of poorly water‐soluble drugs for pulmonary administration. The study revealed that Cur–CD–MOFs showed a considerable improvement in aerodynamic properties compared with conventional micronized Cur. This superior performance was attributed to the distinctive porous structure and lower density of CD–MOFs, which provided Cur–CD–MOFs with better flow characteristics and a larger surface area than conventional micronized powders. The MD simulation study investigated the spatial distribution of Cur molecules in the porous carriers of CD–MOFs. The results indicate that Cur molecules tend to distribute in the lower energy double CD pores in CD–MOFs and form hydrogen bonds with CDs. As more Cur is added, the Cur molecules disperse into different hydrophobic pores instead of concentrating in the same pore. Once all of the hydrophobic pores have been filled, the remaining Cur molecules enter the larger hydrophilic pores and form nanoclusters. The MD simulation results support He et al.’s findings [189] that nanoclustering enhances the solubility and bioavailability of poorly soluble drugs. The spatial distribution of Cur molecules in CD–MOFs ensures efficient loading of a large number of drug molecules. Upon contact with water, the structure of Cur–CD–MOFs is disrupted, promoting the release of Cur from the hydrophobic pores. This leads to the formation of inclusion complexes with CDs, resulting in an enhancement of solubility and release rate. Additionally, the release of nanoclusters further increases drug availability. In conclusion, Cur–CD–MOFs have unique properties that make them viable pulmonary DDSs with significant potential. They are especially useful for treating diseases that require direct drug release in the lungs, such as talc pneumoconiosis. These findings offer new strategies for pulmonary drug delivery of poorly soluble drugs, which may improve the efficacy of relevant therapeutic regimens.

Liu et al. [190] conducted research on the development of novel biophilic and highly porous crystalline materials, known as γ‐CD MOFs (γ‐CDMOFs), for drug carrier applications. These materials are considered ideal for drug carriers due to their highly selective pore structure and biocompatibility. To effectively utilize γ‐CDMOFs as drug release systems, it is crucial to understand their drug retention and release properties. The authors employed MD simulation to predict the apparent dissociation constants (*k*_off_) of prednisolone (PNS) in γ‐CDMOF. Subsequently, *k*_off_ was used to develop a mathematical model that predicts the release profile of PNS in γ‐CDMOF. This information is crucial for the design and regulation of drug release systems. The study results indicate that the mathematical model accurately matched the experimental measurements with a correlation coefficient of 99.92%, demonstrating the precision of the simulation method. Additionally, the study revealed that the distribution of the drug within the γ‐CDMOF particles and its release profile are primarily controlled by the *k*_off_ of the PNS, which utilizes double γ‐CD as the building block of the MOF molecule. This study presents a dependable technique for forecasting and verifying drug release patterns. It can be applied to the investigation of supramolecular systems and more intricate DDSs, and is beneficial for developing more accurate and efficient drug delivery approaches.

Shahabi et al. [191] investigated the impact of external electric fields on the drug molecule 6‐mercaptopurine in peptide‐based MOF (MPF) through MD simulation. The results indicate that the external electric field can significantly modify the interaction strength between drug molecules and MPF, potentially affecting the performance and efficacy of DDSs. When no electric fields are present, drug molecules tend to remain in the porous nanostructures of the MPF due to stronger adsorption. However, the application of an external electric field weakens this interaction, increasing the likelihood of drug molecules being released from the MPF. The study conducted RDF analysis and found that higher electric field strengths resulted in a lower probability of drug molecules being located further away from the surface of the porous nanostructures. Furthermore, this study found that the diffusion of drug molecules around the MPF increased with higher electric field strength, resulting in greater dynamic motion and diffusion coefficients. The drug molecules may be more readily released from the carrier material when an electric field is applied. Their study demonstrates that external electric fields can effectively modulate the release of drug molecules from advanced nanocarriers, such as MPFs. The study highlights the potential of using electric fields as a tool for drug delivery.

Parsaei et al. [101] conducted a series of studies on two structures proposed by Yaghi et al. [192], MOF‐801 and MOF‐808. They reported a successful preparation of MOF‐801 using a simple, cost‐effective, and environmentally friendly synthetic method with ZrCl_4_ as a metal source and fumaric acid as an organic linker. The structure was investigated as a carrier for the anticancer drug 5‐FU. GCMC simulation and RDF analysis revealed that the hydrogen bonding interaction was the key parameter affecting the loading of 5‐FU in MOF‐801. They further investigated the binding of an unmodified MOF‐808 to the antioxidant drug quercetin (QU) [193]. They also studied the surface modification of MOF‐808 using CS–folic acid (CS–FA) polymer. The modified MOF‐808 (referred to as MOF‐808@CS–FA) was compared with the unmodified MOF‐808 in terms of drug loading, drug release properties, targeting ability, and pH‐dependent release behavior. The QU@MOF‐808@CS–FA, after surface modification, demonstrated superior drug loading and pH‐sensitive release behavior, as well as targeted delivery potential. This highlights the significance of organic modifications in enhancing drug delivery performance. Furthermore, GCMC simulation confirmed hydrogen bonding as a major factor in determining the loading of QU in MOF‐808.

To monitor dopamine (DA) levels in body fluids for Parkinson's disease treatment, Xie et al. [194] developed an unlabeled lanthanide MOFs (Ln‐MOFs). Specifically, they used fluorescent MOFs of the rare earth element europium (Eu) combined with α‐CD. The Eu–α‐CD MOF, prepared using the biomineralization method, exhibits a fluorescence response that correlates with the concentration of DA. This indicates that it can be used to measure the concentration of DA based on changes in fluorescence intensity. They employed MD simulation to gain insight into the interactions and thermodynamic dynamics between CD and DA. The highly selective recognition of DA is due to a synergistic interaction between the antennae effect of Eu^3+^ and the specific electron donor of CD nanoparticles. The Ln‐MOFs demonstrated good performance in response to DA in the concentration range of 10^−9^ to 10^−4^ M, indicating its ability to detect DA in relatively low concentrations. To utilize this finding, they designed a visualization test strip using a nitrocellulose membrane. The membrane was chosen for its protein adsorption properties. At room temperature, Eu–α‐CD nanoparticles can be grown in situ on the membrane to construct a test paper. This test paper can semi‐quantitatively detect DA with the naked eye, which is particularly important for the early diagnosis of Parkinson's disease. It is expected to have a positive impact on the early diagnosis and therapeutic monitoring of Parkinson's disease.

Palabıyık et al. [195] conducted a study on the permeability of 60 bio‐MOFs for urea, creatinine, and water, as well as their membrane selectivity for urea/water and creatinine/water separations. The study used GCMC simulation and equilibrium MD simulation to make these predictions. At 310 K and infinite dilution, the methionine–MOF material OREZES exhibits high selectivity for urea/water separation, while the amino‐MOF material BEPPIX shows significant selectivity for creatinine/water separation. The study also found that the main intermolecular interaction force was van der Waals, indicating that nonpolar or weakly polar van der Waals interactions may be critical for the performance of these bio‐MOFs. The study also discovered that taking into account structural flexibility significantly enhanced the membrane selectivity of urea/water. This implies that when designing and modeling membrane separation materials for uremic therapy systems, it is crucial to consider the structural flexibility of MOFs. The study's MD simulation provided insights into the separation mechanisms and performance. However, the biostability and compatibility of these materials need further experimental verification in real organismal environments. Experimental studies are necessary to clarify the effects of biomolecules that bio‐MOFs are subjected to in the blood and in vivo environments, such as protein contamination and biodegradation. The research results provide potential for the creation of new materials for hemodialysis systems and could aid in the development of enhanced artificial kidney systems.

In Liu et al.’s study [88], the impact of variable amplitude heat flow on the capacity of bio‐MOF‐11 carriers to adsorb tetracycline was examined. To evaluate this impact, the researchers employed MD simulation and LAMMPS software to analyze several critical parameters, including MSD, drug particle number, diffusion coefficient, and IE. The results of the study demonstrated that the IE between the drug and the carrier exhibited a significant decrease from −1376.35 to −1549.35 kcal/mol when the heat flux was increased to 0.04 W/m^2^. Concurrently, the penetration number of the tetracycline drug in the bio‐MOF‐11 carrier increased to 606. However, as the heat flux continued to increase to 0.08 W/m^2^, the penetration number decreased again to 520. This result suggests that at moderate heat flux amplitude, the permeability of the drug was improved, probably due to the increased drug binding and mobility. Conversely, at elevated heat flow amplitudes, the decline in penetration number may be attributed to the disruption of the adsorption mechanism by heat, which in turn affects the stability and efficiency of drug delivery. This study underscores the intricate nature of the effects of heat flow on drug delivery efficiency and molecular behavior and underscores the necessity of optimizing heating conditions and system parameters to enhance the overall performance of DDSs based on tetracycline–MOF‐11 interactions.

In the study by Chen et al. [89], the effect of porosity on the adsorption of DOX on bio‐MOF‐11 carrier was investigated via MD simulation. The study investigated the specific effects of different porosities (1, 2, 3, and 5%) on the adsorption behavior and analyzed several parameters, including drug adsorption, MSD, diffusion coefficient, and IE. The findings of the study demonstrated that porosity exerts a substantial influence on both drug adsorption and diffusion. It was observed that an optimal condition was attained at a porosity level of 3%, where the number of diffusing particles within the MOF‐11 increased to 207, signifying high drug adsorption efficiency and effective diffusivity. This study provides a valuable theoretical basis for the optimization of the application of MOF‐11‐based samples in clinical DOX drug transfer, especially in improving drug delivery efficiency.

Taherpoor et al. [95] investigated the adsorption and cotransport properties of transition metal carbide and nitride (MXen) and Cu(II)‐benzene‐1,3,5‐tricarboxylic acid MOF (Cu‐BTC/MOF) as nanocarriers for two drugs, Cur and PTX, using MD simulation. The study evaluated the effect of nanocarrier surface termination states (bare and oxygen termination, denoted as MXN and MXNO) on drug adsorption efficiency. The simulation results indicate that the adsorption efficiency of Cur drugs improved when the MXN surface termination changed from bare to oxygen termination (i.e., MXNO) due to the enhancement of hydrogen bonding. In contrast, the adsorption behavior of PTX did not significantly change on MXNO nanocarriers, suggesting that the oxygen termination state had less impact on PTX adsorption than on Cur adsorption. Furthermore, it was observed that the MXNO–Cu‐BTC/PTX&Cur nanocarrier exhibited the strongest IE with Cur, while the IE between PTX and the MXNO–Cu‐BTC adsorbent was lower in the MXNO–Cu‐BTC coloading system. This implies that the interaction energies between different drugs and adsorbents may be influenced by their respective structures. The study investigated the interactions between drugs, water molecules, and nanocarriers in the system. The intensity of the RDF peaks confirmed the stronger interactions present in the coloading system. Additionally, the study examined the IE between the drug and water, as well as its variation with time during the simulation. The initial IE between PTX and water was higher in the MXN system. However, it decreased significantly after equilibrium. This suggests that as the system equilibrates, PTX tends to decrease its interaction with water molecules and increase its interaction with the nanocarriers. In summary, the results confirm that the surface termination states of the MXN and MXNO–Cu‐BTC structures observed in MD simulation have a significant impact on the adsorption and codelivery behavior of the drugs Cur and PTX. These findings provide a preliminary understanding of the potential of these nanocarriers as efficient DDSs and are important references for future medical experiments and development of DDSs using these nanocarriers.

In brief, recent MD studies on emerging MOF families, including bio‐MOFs, COFs, and hybrid composites, highlight the expanding scope of MOF‐based DDSs beyond conventional crystalline frameworks. These systems exhibit enhanced biocompatibility, hierarchical porosity, and increased adaptability at host–guest interfaces, offering new opportunities for the development of multifunctional drug carriers.

Comparative investigations across different MOF families further indicate that framework composition, topology, and metal ligand chemistry collectively determine the balance between structural stability, drug affinity, and release behavior. Distinct advantages have been identified among representative families, such as the structural simplicity of IRMOFs, the pH sensitivity of ZIFs, the coordination tunability of MIL‐type frameworks, and the exceptional robustness of UiO‐based systems. Together, these family specific studies establish an empirical foundation for understanding how MOF chemistry and structure influence drug delivery performance.

Beyond computational analyses, emerging experimental and preclinical studies have begun to assess MOF‐based platforms in animal models, providing early evidence on biodistribution, therapeutic efficacy, and safety profiles. These reports consistently demonstrate that in vivo behavior is highly dependent on framework composition, particle size, surface chemistry, dose, and administration route, underscoring the importance of integrating simulation insights with experimental validation.

Taken together, comparative studies across different MOF families highlight that framework topology, chemical composition, and structural flexibility collectively govern drug–MOF interactions and release behavior under biologically relevant conditions. While individual MOF platforms exhibit distinct advantages, these observations underscore the necessity of a unified molecular‐level perspective to rationalize how structural features translate into functional performance in drug delivery. Building upon the family‐specific insights summarized above, the following section integrates MD simulation results to distill general mechanistic principles governing drug loading, retention, release, and biological interactions in MOF‐based DDSs.

## 6 From Molecular Insights to Design Principles and Clinical Translation

While the preceding sections focus on specific MOF families and representative application scenarios, MD simulation offers an opportunity to move beyond case‐by‐case observations toward a more general mechanistic understanding of MOF‐based DDSs. By resolving drug–framework interactions, structural fluctuations, and environmental responses at atomic resolution, MD simulation enables the identification of recurring physicochemical determinants that govern drug‐loading efficiency, stability, and release behavior across diverse MOF platforms. In this section, we consolidate these molecular‐level insights to establish a coherent framework that links structural features and interaction mechanisms with functional performance, thereby providing a mechanistic foundation for interpreting experimental observations and evaluating MOF behavior under different biological conditions.

### 6.1 Deriving Molecular Insights From MD Simulations

MD simulation provides fundamental insights into the molecular mechanisms governing drug loading, retention, and release in MOFs, thereby establishing a theoretical basis for the rational design and selection of MOF‐based DDSs. Among the key factors influencing drug‐loading behavior, geometric compatibility between drug molecules and the pore architecture of MOFs plays a central role. Previous simulation studies have demonstrated that pore size, shape, and connectivity strongly affect drug diffusion pathways, loading capacity, and spatial distribution within the framework. When pore dimensions are comparable to the molecular size of the drug, enhanced confinement effects and prolonged residence times are commonly observed, whereas excessively large pores often result in reduced effective confinement and accelerated release behavior [114].

Beyond geometric considerations, specific intermolecular interactions between drug molecules and MOF surfaces decisively influence adsorption strength and release kinetics. MD simulation has shown that functional groups on organic linkers and metal nodes can participate in hydrogen bonding and electrostatic interactions with drug molecules, thereby modulating interaction energies, diffusion barriers, and residence times [94]. These molecular‐level interaction mechanisms provide a clear rationale for experimentally observed variations in drug‐loading efficiency and release profiles arising from framework functionalization.

Framework flexibility further represents a critical determinant of drug delivery behavior, particularly for MOFs exhibiting structural responsiveness under physiological conditions. Dynamic structural fluctuations can transiently alter pore accessibility and modulate drug mobility within the framework, thereby affecting diffusion coefficients and release kinetics. In addition to intrinsic structural features, environmental parameters, including solvent composition, pH, ionic strength, and temperature, exert pronounced effects on drug–MOF interactions and framework stability. MD simulation enables these factors to be explicitly incorporated, clarifying their roles in solvation, competitive adsorption, and framework integrity under biologically relevant conditions [114]. Collectively, these insights demonstrate that MD simulation is a powerful tool for establishing structure–property relationships and guiding the rational optimization of MOFs for efficient, stable, and responsive drug delivery applications.

In addition to host–guest interaction energetics, MD simulation has highlighted the importance of framework lability and degradation pathways in determining the biological fate of MOF‐based DDSs. Hydrolytically labile frameworks, such as certain zinc‐based ZIFs, may undergo accelerated bond weakening and partial structural collapse under acidic or enzymatic conditions, leading to the release of metal ions and linker fragments that influence both drug release behavior and clearance profiles [102, 196]. In contrast, zirconium‐based UiOs generally exhibit slower degradation kinetics and higher structural robustness, which can mitigate acute toxicity but may also result in prolonged retention if clearance pathways are limited [197].

Furthermore, interactions with biologically relevant macromolecules, including proteins and lipids, play a crucial role in shaping the in vivo performance of MOF nanocarriers. Molecular simulation, supported by complementary experimental observations, indicate that surface chemistry strongly affects protein corona formation, immune recognition, and subsequent biodistribution. Surface modification strategies, such as polymeric or polysaccharide coatings, have been shown to reduce immune activation and enhance biocompatibility by modulating interfacial interactions at the nano‐bio interface [198]. These findings underscore that MD simulation provides mechanistic insight not only into drug loading and release but also into degradation behavior and early‐stage biological interactions.

While these molecular insights substantially advance our understanding of MOF‐based drug delivery mechanisms, their predictive power remains constrained by the intrinsic limitations of conventional MD simulation, particularly with respect to accessible time scales, length scales, and model transferability. Addressing these limitations is therefore essential for extending molecular‐level understanding toward quantitative prediction and rational material screening.

### 6.2 Translating Molecular Insights Into Design Principles

Translating molecular‐level insights into practical design strategies represents a critical step toward rational MOF engineering for drug delivery applications. The mechanistic understanding derived from MD simulation enables the formulation of design principles that link MOF physicochemical properties to functional performance metrics, including loading capacity, release kinetics, and structural stability.

For instance, simulation studies suggest that drugs with high polarity or strong hydrogen‐bonding propensity preferentially interact with frameworks featuring polar linkers or accessible metal coordination sites, whereas hydrophobic drugs benefit from confinement within nonpolar pore environments that enhance residence times [94, 114]. Similarly, frameworks with moderate flexibility can facilitate controlled release by balancing confinement with dynamic pore accessibility, while overly rigid structures may impede efficient release under physiological conditions [25, 163]. Environmental responsiveness, such as pH‐triggered structural lability, further provides a means to tailor release profiles for site‐specific drug delivery [102].

By mapping simulation‐derived descriptors, such as interaction energies, residence times, and diffusion coefficients, onto performance objectives, MD‐guided strategies offer a systematic framework for selecting and optimizing MOF architectures tailored to specific therapeutic requirements. These principles highlight the role of molecular simulation as a design‐oriented tool rather than a purely descriptive technique.

### 6.3 Bridging MD‐Guided Design to Clinical Translation

MD simulation has established a solid mechanistic foundation for understanding drug loading, release, and stability in MOF‐based DDSs. Recent studies demonstrate that MOF surfaces exhibit appreciable affinity for biomolecules, including representative nucleic acids and proteins, and that electrostatic interactions, hydrogen bonding, and hydrophobic effects collectively govern adsorption behavior and interfacial stability. These findings provide a molecular basis for evaluating MOF biocompatibility and early‐stage nano‐bio interactions [104, 133, 198].

In parallel, simulation studies focusing on stimuli‐responsive drug release and framework stability have offered valuable insights into how MOF structures respond to physiologically relevant environments, such as acidic conditions and variable ionic strength. These investigations indicate that changes in local coordination environments can alter framework integrity, guest mobility, and degradation tendencies, thereby influencing retention and release profiles under biological conditions [114, 136, 187]. Comparative analyses across different MOF families further suggest that variations in framework composition and surface chemistry are key determinants of stability and degradation behavior in aqueous and biologically relevant media [198].

Despite these advances, a major obstacle to direct clinical translation lies in the limited ability of conventional MD simulations to quantitatively describe long‐term degradation kinetics and biological fate under realistic physiological conditions. In vivo environments involve complex mixtures of plasma proteins, enzymes, ions, and dynamic biochemical processes that exceed the accessible time and length scales of atomistic simulations. Moreover, the predictive accuracy of existing force fields remains insufficient for reliably modeling reactive events central to biological fate, including metal–ligand bond cleavage and protein‐mediated degradation pathways [199].

To overcome these challenges, emerging computational strategies are expected to play a central role in bridging the clinical translation gap. Multiscale simulation frameworks that integrate atomistic and coarse‐grained descriptions can extend spatial and temporal coverage, while machine‐learning‐assisted potentials offer new opportunities to improve simulation accuracy and transferability for complex interfacial interactions and reactive behavior [44, 60]. In addition, data‐driven modeling approaches that integrate simulation outputs with experimental datasets may enable quantitative prediction of protein–MOF interactions, degradation pathways, and in vivo fate, thereby supporting the rational design and translational development of MOF‐based DDSs [200, 201].

## 7 Beyond Conventional MD: Multiscale Simulations and ML

**FIGURE 5:**
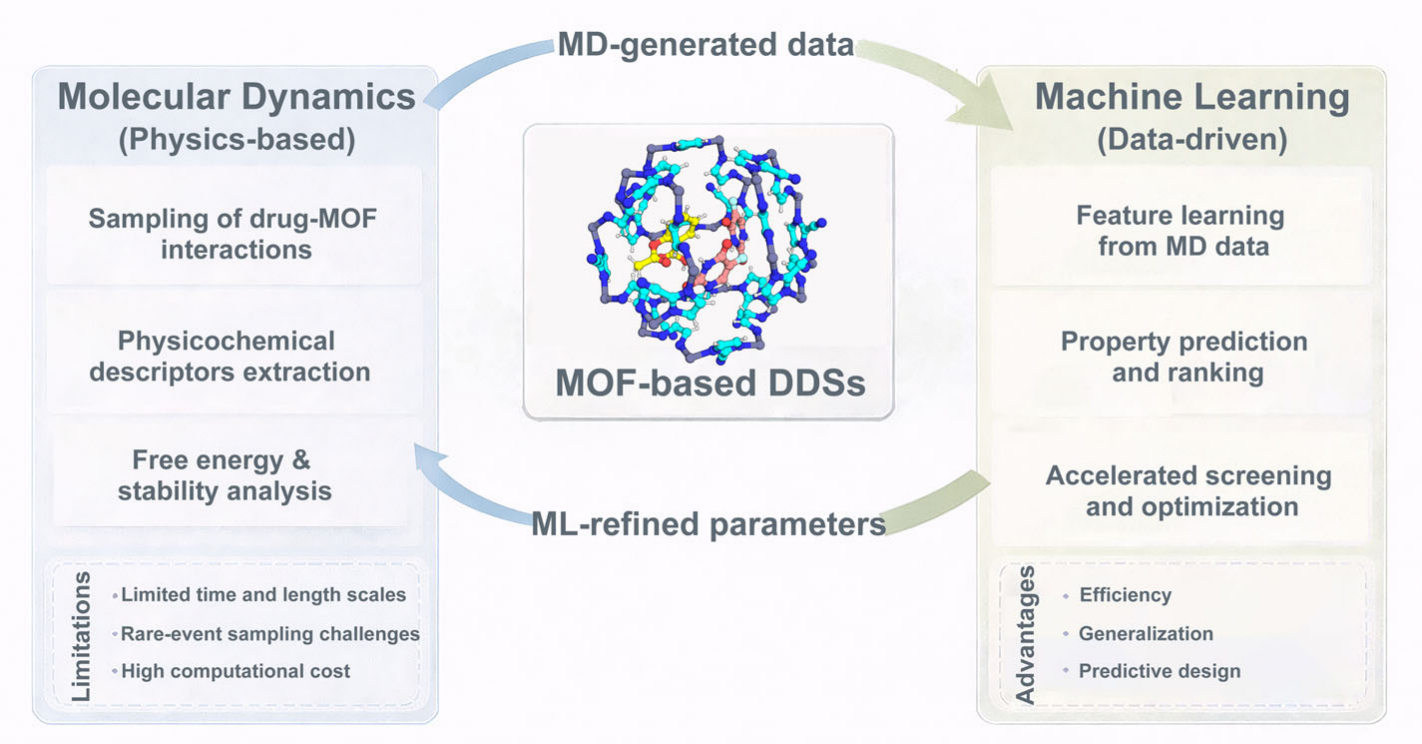
Closed‐loop framework integrating molecular dynamics and machine learning for MOF‐based drug delivery systems. Physics‐based MD simulations generate atomistic trajectories and physicochemical descriptors that are used for feature learning and property prediction in machine learning models, while ML‐refined parameters and screening results feed back into simulation design, forming an iterative strategy for accelerated optimization and rational development of MOF‐based DDSs.

Despite the valuable mechanistic insights provided by atomistic MD simulation, capturing the full complexity of MOF‐based DDSs under realistic biological conditions remains challenging. In particular, degradation processes, immune interactions, and long term biodistribution involve time and length scales that exceed the practical limits of conventional MD simulation [200]. As a result, direct simulation of in vivo relevant phenomena, such as protein corona evolution, framework breakdown, and clearance pathways, remains computationally prohibitive. As shown in Figure 5, MD simulation serves as a physics‐based engine to sample drug–MOF interactions, extract key physicochemical descriptors, and quantify free energy landscapes and stability profiles. These MD‐generated data provide high‐quality training inputs for ML models, which enable feature learning, property prediction, and accelerated screening of MOF candidates. In turn, ML refines simulation parameters and guides targeted MD studies, forming an iterative feedback loop that enhances efficiency, generalizability, and predictive capability. This integrated MD‐ML workflow offers a scalable computational paradigm for bridging atomistic understanding and data‐driven optimization in MOF‐based drug delivery research.

To bridge this gap, future simulation driven design strategies should integrate degradation sensitive models with targeted experimental validation. Coupling atomistic predictions with preclinical in vivo studies can help establish feedback loops that refine force field parameterization, validate degradation pathways, and improve the predictive reliability of computational screening [199, 202]. Such an integrated simulation experiment framework is essential for translating molecular insights into practical guidelines for safe and effective MOF‐based DDSs.

### 7.1 Multiscale Modeling Approaches

Multiscale modeling provides a hierarchical framework linking different resolutions of molecular representation. Atomistic MD simulation captures local interactions and hydrogen‐bonding dynamics, whereas CG models extend temporal and spatial coverage to the microsecond and micrometer regimes. For MOF‐based DDSs, hybrid QM/molecular mechanics (MM) schemes have been applied to describe coordination bonding between metal centers and drug molecules with chemical accuracy [203, 204], while CG simulations are employed to probe macroscopic release kinetics and aggregation in physiological environments [205, 206, 207]. Such integration allows researchers to connect local adsorption sites with macroscopic drug transport, offering a holistic understanding of stimuli‐responsive release mechanisms. Additionally, coupling MD with continuum models or kinetic MC (kMC) simulations facilitates the quantitative prediction of long‐term drug diffusion and degradation behavior within complex media such as plasma or tumor microenvironments.

### 7.2 Integration of ML

ML is rapidly transforming the landscape of MOF simulation and design, bridging data‐driven inference with atomistic modeling. As summarized by Park et al. [201], ML methods have been deployed across multiple layers of MOF research, ranging from structure–property regression models and generative design to the development of ML interatomic potentials (MLPs). MLPs, such as those trained via neural network or Gaussian approximation potentials, can reproduce quantum‐level accuracy at near‐classical MD cost, enabling longer simulations of drug–MOF interactions with dynamic framework flexibility. These models have been successfully demonstrated in ZIFs and amorphous MOFs, allowing efficient exploration of diffusion and adsorption processes that are beyond the reach of traditional force fields [208].

Furthermore, ML has been instrumental in high‐throughput virtual screening of MOFs for biomedical applications. As Melle et al. [209] demonstrated, hierarchical workflows integrating ML classifiers with molecular simulations can predict MOF biocompatibility and drug‐loading capacity, drastically reducing the experimental search space. For instance, random forest and graph neural network (GNN) [210] models have been used to evaluate metal toxicity, linker safety, and pore accessibility, achieving > 80% accuracy in biocompatibility prediction [211]. Combined with grand canonical GCMC and MD simulation, this pipeline provides a quantitative basis for the rational selection of candidate MOFs such as Zr‐ and Fe‐based systems for in vitro and in vivo studies.

Emerging generative models, including variational autoencoders [212] and diffusion models [213], further extend the predictive design frontier by creating hypothetical MOF structures with tailored physicochemical properties. These AI‐driven design strategies are gradually being integrated into autonomous laboratories that combine robotic synthesis with Bayesian optimization, establishing a self‐driving loop between ML prediction, experimental validation, and simulation refinement [214]. Such integration points toward a future where multiscale MD and ML synergistically enable predictive, data‐guided, and adaptive modeling of MOF‐based DDSs, closing the loop between computation and experiment.

## 8 Challenges, Future Perspectives, and Conclusion

### 8.1 Current Challenges

Despite the rapid development of MD simulation in understanding MOF‐based DDSs, several challenges still limit their predictive accuracy and translational relevance. First, the transferability of existing force fields remains a major obstacle. Most conventional biomolecular force fields (e.g., AMBER, CHARMM, OPLS‐AA) inadequately describe the coordination environment of metal centers, while MOF‐specific force fields (e.g., UFF4MOF, MOF‐FF) often fail to capture the flexibility and partial covalency of the framework. Parameterization for hybrid MOF‐biomolecule systems therefore remain laborious and system‐specific.

Second, time‐ and length‐scale limitations hinder the exploration of slow or rare events such as framework breathing, hydrolytic degradation, or long‐term drug diffusion across interfaces. Although coarse‐grained and enhanced sampling techniques extend accessible scales, the loss of atomistic resolution or the difficulty in parameter validation still restricts their application.

Third, simulating biologically relevant conditions, such as ionic strength, protein corona formation, and physiological degradation, poses additional complexity. Most simulations are still performed in idealized environments, far from the heterogeneous biological milieu encountered in vivo. Bridging this gap requires accurate representation of solvent effects, ion distributions, and dynamic surface chemistry.

Finally, reproducibility and benchmarking remain underdeveloped. Differences in model setup, protonation state assignment, and simulation protocols can lead to inconsistent results, emphasizing the need for standardized simulation workflows and publicly available datasets.

### 8.2 Future Perspectives

The future of MOF‐DDSs simulations lies in integration, automation, and prediction. First, multiscale frameworks combining quantum mechanical accuracy with coarse‐grained efficiency are expected to provide a seamless connection between local coordination chemistry and macroscopic drug transport. Hybrid QM/MM approaches will help reveal degradation and functionalization mechanisms, while continuum diffusion modeling can translate microscopic mobility data into experimentally measurable release profiles.

Second, ML is poised to redefine MD‐based material discovery. MLPs can retain near‐ab initio accuracy while drastically reducing computational cost, enabling long‐timescale simulations of flexible and defect‐rich MOFs. Meanwhile, high‐throughput screening guided by GNNs or random forest classifiers can identify optimal MOFs for biocompatibility, drug loading, and stability, as demonstrated in recent studies.

Third, integrating ML‐driven prediction with autonomous experimentation may close the loop between simulation and synthesis, creating a self‐evolving design framework for MOF‐based nanocarriers. Such data‐centric pipelines will accelerate the translation of computational insights into experimentally validated drug delivery platforms.

Finally, as simulation and experimental resolution converge, future studies may extend beyond conventional cytocompatibility to probe interfacial interactions with bio‐membranes, enzymatic degradation, and dynamic host–guest exchange, thereby connecting atomic‐scale mechanisms with therapeutic efficacy.

## 9 Conclusion

In summary, this review systematically discussed the application of MD simulation in elucidating the adsorption, diffusion, release, and stability mechanisms of MOF‐based DDSs. By integrating atomistic insight with materials design, MD simulation has become a crucial bridge between experimental observation and theoretical understanding.

The emerging convergence of multiscale modeling and ML is expected to transform the field from descriptive analysis toward predictive and design‐oriented simulations, guiding the rational development of next‐generation MOF carriers with controllable drug release, enhanced stability, and improved biocompatibility.

Overall, MD simulation serves not merely as a computational complement but as a driving force in advancing MOF‐based DDSs toward precision medicine and clinical translation.

## Author Contributions

Jiahao Xu: conceptualization, data curation, formal analysis, and writing – original draft. Hanzi Zheng: formal analysis, validation, and writing – review and editing. Yue Gao: data curation and validation. Yuanqiu Lai: software and visualization. Mengya Peng: validation and writing – review and editing. Yike Hu: writing – review and editing. Tianmeng Yuan: writing – review and editing. Xiang Liu: writing – review and editing. Shihan Zhou: writing – review and editing. Wei Duan: conceptualization, supervision, funding acquisition, project administration, and writing – review and editing. Jia–Wei Shen: conceptualization, supervision, funding acquisition, project administration, and writing – review and editing. Yongke Zheng: conceptualization, supervision, project administration, and writing – review and editing. All authors have read and approved the final manuscript.

## Ethics Statement

The authors have nothing to report.

## Conflicts of Interest

The authors declare no conflicts of interest.

## Data Availability

The authors have nothing to report.
